# The tongue microbiome of young patients with chronic kidney disease and their healthy mothers

**DOI:** 10.1007/s00784-024-05492-x

**Published:** 2024-01-24

**Authors:** Karolin C. Hoefer, Lutz T. Weber, Anna Greta Barbe, Isabelle Graf, Stefanie Thom, Angela Nowag, Claus J. Scholz, Hilmar Wisplinghoff, Michael J. Noack, Nathalie Jazmati

**Affiliations:** 1 University of Cologne, Faculty of Medicine and University Hospital Cologne, Polyclinic for Operative Dentistry and Periodontology, Cologne, Germany; 2 Children´s and Adolescents Hospital, Pediatric Nephrology, University Hospital of Cologne, Faculty of Medicine, University of Cologne, Cologne, Germany; 3 Department of Orthodontics, Faculty of Medicine and University Hospital of Cologne, Cologne, Germany; 4 Wisplinghoff Laboratories, Cologne, Germany; 5 Institute for Virology and Microbiology, Witten/Herdecke University, Witten, Germany; 6 Institute for Medical Microbiology, Immunology and Hygiene, University of Cologne, Cologne, Germany

**Keywords:** Adolescents, Child, Microbiome, Mothers, Chronic kidney disease, Tongue

## Abstract

**Objectives:**

Oral microbiome plays a crucial role in the incidence and development of oral diseases. An altered intestinal microbiome has been reported in adults with chronic kidney disease (CKD). This study aimed to characterize the tongue microbiome of young patients with CKD compared to their healthy mothers to identify the influence of CKD-associated factors on resilient tongue ecosystem.

**Material and methods:**

Thirty patients with CKD (mean age, 14.2 years; 16 males and 14 females) and generalized gingivitis were included in the study. Swabs of the posterior tongue were collected from the patients and 21 mothers (mean age 40.8 years). Next-generation sequencing of 16S rDNA genes was employed to quantitatively characterize microbial communities.

**Results:**

The bacterial communities were similar in terms of richness and diversity between patients and mothers (*p* > 0.05). In patients with CKD, 5 core phyla, 20 core genera, and 12 core species were identified.

**Conclusions:**

The tongue microbiome of the study participants showed no relevant CKD-associated differences compared to their mothers and appears to be a highly preserved niche in the oral cavity. Differences observed in the abundance of individual species in this study could be attributed to the age rather than CKD, even after a mean disease duration of 11 years.

**Clinical relevance:**

CKD and its associated metabolic changes appear to have no detectable impact on the resilient tongue microbiome observed in young patients.

**Supplementary Information:**

The online version contains supplementary material available at 10.1007/s00784-024-05492-x.

## Introduction

Children and adolescents with chronic kidney disease (CKD) have increased comorbidities and vulnerabilities. In Europe, approximately 11–12/million children and adolescents develop stage 3–5 chronic kidney disease (CKD) annually; the prevalence is estimated at 60–70/million in the age-related population [1]. The prevalence of CKD is approximately two times greater in boys owing to their higher disposition to urinary tract malformations as compared to that in girls [2]. The type and severity of primary kidney disease determine the course of kidney failure.

CKD in adults is associated with an imbalance in the human intestinal microbiome. This imbalance could be attributed to contributory CKD-associated factors such as uremia, increased inflammation and immunosuppression, as well as pharmacological therapies, and dietary restrictions [3]. Furthermore, various therapies in patients with CKD, such as hemodialysis or peritoneal dialysis, may be linked to variations in the intestinal microbiome [3].

The key research topic in this study was whether alterations in the microbiome owing to CKD are also prevalent in the oral cavity of younger patients. The homeostasis of the human microbiome is highly dependent on environmental conditions. Therefore, this complex system is highly influenced by health, disease conditions, and the therapeutic strategies applied [3]. Alternative therapies or disease duration could be responsible for the differences between adult and young patients with CKD. Limited published data on the development of the microbiome in children and adolescents with CKD are available [4].

The oral cavity, which is a unique ecosystem marking the beginning of the gastrointestinal tract, represents the second largest microbial community in humans [5]. With its anatomical and diverse oral niches, the oral cavity is inhabited by oral microbiota of more than 700 widespread taxa [6, 7]. The tongue microbiome is one of the most resilient niches within the oral cavity and has been regularly analyzed in clinical studies [6]. Oral microbiota, including bacteria, viruses, archaea, and fungi, are known to cause caries and periodontitis, which are the two most common oral diseases. In addition, oral microbiota is considered a significant risk factor for systemic health conditions in humans, such as diabetes mellitus, cardiovascular disease, and bacteremia, and premature and low birth weight infants [8, 9]. For a better understanding of colonization of bacterial species and development of mature microbiota in the oral cavity, it is important to consider oral microbiota development during infancy [10].

The microbiota of a newborn is highly dynamic and changes rapidly in its composition, especially during the first years of life, towards a stable adult-like structure that includes diverse microbial communities with unique composition and functions at specific body sites [11]. Colonization of the oral mucosal surfaces starts during birth with the introduction of bacteria and fungi through multiple pathways, including maternal transmission during childbirth, parental exposure, nutrition, and horizontal transmission through caregivers and peers. A microbial community is established by the eruption of teeth into a diverse microbiome [12–14]. A complex interplay exists between the establishment and development of neonatal immunity, and early microbial acquisition [15]. These early life interactions between the microbiome and human, in particular in a household, host are responsible for the characteristics of postnatal acquired and innate immune functions and physiological development influencing future health [11, 16–18].

Metabolic changes imposed by CKD, inherited genetic factors, and immunocompetence transmitted from the mother affect the development of the oral microbiome in children with CKD.

Therefore, this study aimed to characterize the tongue microbiome as a resilient niche in the oral cavity of children, adolescents, and young adults with CKD, and compare it to that of their mothers.

## Material and methods

### Patients and study design

This cross-sectional study aimed to characterize the tongue microbiome of children with renal diseases and compared it with that of their healthy mothers.

The trial was approved by the Ethics Committee of the Faculty of Medicine, University of Cologne, Germany, and recorded at The German Clinical Trials Register (registration number DRKS00010580).

### Sample size calculation

To estimate the required study sample size, we used the R package pwr. To detect differential relative abundances among 20 bacterial taxa between patients and their mothers with two-tailed *t*-tests at a Bonferroni-corrected significance level of 5% and a power of 80%, the sample size requirements were based on the expected effect size. Assuming Cohen’s small effect size, 750 individuals per group were required; medium-sized effects required *n*=122 per group, and large effects could be detected with 50 participants per group. The present study sample of 30 patients and 21 mothers provided a power of 36% to detect large effects in the abovementioned setting.

### Patient recruitment

The enrolled patients represented a typical study population of the Department of Pediatric Nephrology at the University Hospital of Cologne. A total of 30 participants, who appeared for their CKD control assessment from July 1, 2016, to 2019 were consecutively included in the study. All the patients were initially enrolled by a pediatric nephrologist and examined by the dentists involved in this study. Healthy mothers of the study participants were included as controls. All mothers that were included as controls were living in the same household with the respective CKD patient. The initial study design included healthy siblings as a control group. This was also intended to investigate the family connection. Nevertheless, a large part of the siblings also suffered from a chronic disease. Due to the general illnesses, the siblings were no longer available as a control group.

### Inclusion and exclusion criteria

Patients who regularly attended the Department of Pediatric Nephrology at the University Hospital for examination were screened by pediatric nephrologists according to the following criteria: patients with CKD grades 1 to 5 according to the Kidney Disease Improving Global Outcomes (KDIGO) classification [19], who were conservatively treated, underwent transplantation or dialysis, and those with gingivitis. The patients were subsequently examined by the study dentist; a gingival index (GI) > 0 and periodontal screening index (PSI) of 0–2 for oral health evaluation were required. The exclusion criteria were any signs of acute infection, fever, or antibiotic treatment 14 days prior to participation. This decision was made by pediatric nephrologists based on the clinical parameters and blood tests. Prior to participation in the study, written informed consent was obtained from the parents/legal guardians of young patients eligible for the study and if indicated, from the participants themselves.

### Study measures

The age, sex, and gender of all the patients, as well as the underlying disease, time of diagnosis, dialysis (in years), treatment measures, and medication intake were evaluated. Swabs of the tongue were collected from each patient and from their mothers in this cross-sectional study to analyze and compare the oral microbiome. The main clinical parameters investigated in the patient group for determining oral inflammation were the Papillary Bleeding Index (PBI), Quigley–Hein Index (QHI), and Approximal Plaque Index (API). The dentition status was recorded in all the patients.

### Microbiome sampling

Tongue swabs were obtained in the morning, and participants were asked to refrain from brushing their teeth or using mouthwashes for 12 h before the sample was taken. Oral microbiome samples were collected from the posterior tongue dorsum using dry cotton swabs (Microbrush, Germany), transferred to sterile 1.5-ml reaction tubes (Eppendorf, Germany), and stored at −80 °C until use. Sampling was performed according to the protocol of Zaura et al. [20]. DNA was isolated using a QIAMP DNA Mini Kit (Qiagen, Germany) according to the manufacturer’s instructions. All extracted DNA samples were quantified using Qubit dsDNA kit (Thermo Fisher Scientific, MA, USA) and NanoDrop (Thermo Fisher Scientific, MA, USA) for sufficient quantity and quality of input DNA for 16S sequencing. Furthermore, amplicons were cleaned before library preparation using the NucleoMag NGS Clean-up (Macherey-Nagel, Germany).

### 16S rRNA gene sequencing

DNA samples were processed with the Ion 16S Metagenomics Kit (Thermo Fisher Scientific, Germany) using two primer pools, thereby amplifying seven of the nine hypervariable bacterial 16S rDNA regions (pool 1: V2, V4, and V8; pool 2: V3, V6/7, and V9). Amplicons were pooled and cleaned using the NucleoMag NGS Clean-up (Macherey-Nagel, Germany), followed by library preparation using an Ion Plus Fragment Library Kit (Thermo Fisher Scientific, Germany). Metagenomic DNA libraries were sequenced on an Ion Torrent platform using S5 and S5 Prime devices (Thermo Fisher Scientific, Germany).

### Raw data analysis

Primary data analysis was tailored to the generation of microbiome profiles from raw sequencing reads and performed in the Qiime2 (2021.4 core distribution) environment. For read quality control, the nucleotides following sequences of three low-quality (PHRED score less than 20) base calls were eliminated, and only the read was retained in the analysis if at least 50% of the nucleotides remained after truncation. Thereafter, the residual sequences of library adapters (5′- ATCACCGACTGCCCATAGAGAGGCTGAGAC-3′) were eliminated, requiring a minimum remaining read length of 150 nucleotides. The reads were subsequently subjected to denoising and dereplication using dada2 in denoise-pyro mode with parameters --p-trim-left 20 and --p-trunc-len 0. The resulting representative amplicon sequences were then assigned to 99% sequence similarity-clustered SILVA v138 taxonomies using vsearch with parameters --p-maxaccepts 25, --p-perc-identity 0.97, and --p-strand 'both'. SILVA v138 taxonomies that were restricted to the domain of bacteria and where species names did not contain “uncultured” or “metagenome.”

### Bioinformatical analysis

Secondary data analysis involved bioinformatics and statistics to describe and visualize the microbiome dataset, which was performed in the R environment using diverse data science packages. For analysis at the phylum, genus, and species, postprocessing was limited to the exclusion of all reads that were assigned lower than the family level (assuming low-quality reads that did not reach the family level). To analyze and describe the core operational taxonomic units (OTUs) that were identified down to the species level, we applied more stringent postprocessing criteria: low relative abundance (f<2.5%) and rare species (occurrence in less than 10 samples) were excluded from the analysis. To ensure saturation of the species-level microbiome profiles, we performed rarefaction analysis and excluded all the patients (and their corresponding mothers) who did not contain sufficient reads after postprocessing (Online Resource 1). We excluded five samples (*CKD 30*, *CKD 32*, *Mother 30*, *Mother 31*, and *Mother 32*) that did not reach saturation. The sequencing depth of all other samples was sufficient to identify the bacterial community members of each individual microbiome at the species level and was therefore included in this analysis.

Radar plots were generated using R package ggradar. Alpha diversity was assessed using the Shannon diversity index (− ∑ *p* × *log*_2_*p*) at the genus and species levels. For rarefaction analysis, we first calculated a blueprint profile containing the species averages across the study population; the rounded products of the target read depths using this blueprint were then utilized to calculate alpha diversities. Beta diversity, that is, the distance between the microbiome profiles, was determined using the weighted UniFrac method [21]. Differentially abundant taxa were determined using two-sided Welch two-sample *t*-tests on relative abundances; false discovery rates were calculated using the Benjamini-Hochberg method to correct for multiple testing. False discovery rates (FDR) < 0.05 were considered significant.

### Data availability

Sequencing data and sample metadata are available at the Sequence Read Archive (https://www.ncbi.nlm.nih.gov/sra) under accession number PRJNA938485.

## Results

### Description of the study population

Thirty participants with CKD were enrolled. Sixteen male and 14 female participants between 6 and 25 years of age (mean ± standard deviation [SD]; 14.2±5.2) were included in the study; we included 9 patients ≥ 18 years who were still undergoing pediatric nephrology treatment (transitional phase). The patients were in a physically and/or mentally retarded condition at this time and definitely benefited from pediatric care, despite being chronologically adult. This individual relevance of prolongation of the transitional phase beyond the age of 18 is described by the professional society for transitional medicine. In our study, 13 and 17 participants were in the mixed and permanent dentition phase, respectively. The primary diseases of the patients were as follows: congenital anomalies of the kidney and urinary tract (CAKUT), 11; glomerulopathy, 9; ciliopathy, 7; systemic disease, 1; and other diseases, 2. The patients underwent the following therapies: conservative, 7; dialysis, 2; and transplant, 21.

In our study group, the mean disease duration was 11 years (range, 1–22 years). Only 2 of the 30 patients (6.7%) underwent dialysis for 1 and 6 years, respectively (Table 1).

**Table 1:** Baseline demographic and clinical characteristics of the study group

	Total (*n*=30)
Sex	
Males	16 (53.3%)
Females	14 (46.7%)
Age in years	
Mean ± SD	14.2±5.2
Range	6-26
DMFT/dmft	
Mean ± SD	0.6±1
PBI	
Mean ± SD	1.1±0.7
QHI	
Mean ± SD	2.5±0.9
API	
Mean ± SD	90±18.9
Dentition	
Mixed	13 (43.3%)
Permanent	17 (56.7%)
Primary disease	
*CAKUT*	11 (36.7%)
*Glomerulopathy*	9 (30%)
*Ciliopathy*	7 (23.3%)
*Systemic disease*	1 (3.3%)
*Renovascular*	0 (0%)
*Others*	2 (6.7%)
Therapy	
*Conservative*	7 (23.3%)
*Dialysis*	2 (6.7%)
*Post-transplant*	21 (70%)
Duration of CKD disease in years (mean (max))	11 (max 22)
Dialysis Duration (years)	2 patients 1 and 6
Medication	
*Immunosuppression*	22 (73.3%)
*Including cyclosporin*	5 (16.7%)
*Amlodipine*	17 (56.7%)
*Ramipril*	11 (36.7%)
*Amlodipine + ramipril*	4 (13.3%)

*CAKUT* congenital anomalies of the kidney and urinary tract, characteristics for categorical variables are presented as *n* counts and (percentage); continuous variables as mean ± standard deviation (SD)

In addition to the 30 young participants with kidney disease, the mothers of the enrolled patients were offered a tongue swab to participate in the microbiome analysis as controls. Twenty-one mothers agreed to participate in the study and provided consent. The mothers had no recorded history of chronic diseases, especially CKD. All the 21 participating controls underwent regular dental checkups with their dentists and were not diagnosed with periodontitis and asked about their oral health history. Anamenestically, there was no evidence of oral/mental disease entities in any mother. The mean age of the mothers was 41 years (mean ± SD; 40.8±6.5). The baseline demographic and clinical characteristics of the study groups are presented in Table 1.

### Intraoral findings

The enrolled participants showed generalized gingivitis (mean PBI score ± SD; 1.1±0.7), which was present at all sites, both buccal and lingual mucosa; however, the caries experience was low with the Decayed, Missing, and Filled permanent/or deciduous Teeth (DMFT/dmft) value (mean± SD; 0.6±1) being predominantly filled teeth. The measured biofilm values on the buccal and lingual sides according to QHI (mean ± SD; 2.5±0.9) showed a relevant amount of plaque.

### Microbiome analysis

First, the sequencing data of 30 patients in the CKD group and 21 healthy mothers in the control group were analyzed and compared to describe the microbiome characteristics of the tongue at the phylum and genus levels. The bacterial taxonomic composition revealed 12 different phyla in the study group. Of these, only five (core) phyla (*Firmicutes*, *Proteobacteria*, *Bacteroidota*, *Actinobacteria*, *Fusobacteria*) were present with > 1% on average and accounted for over 98% in the study group as well as in the control group (Online Resource 2).

**Fig. 1:**
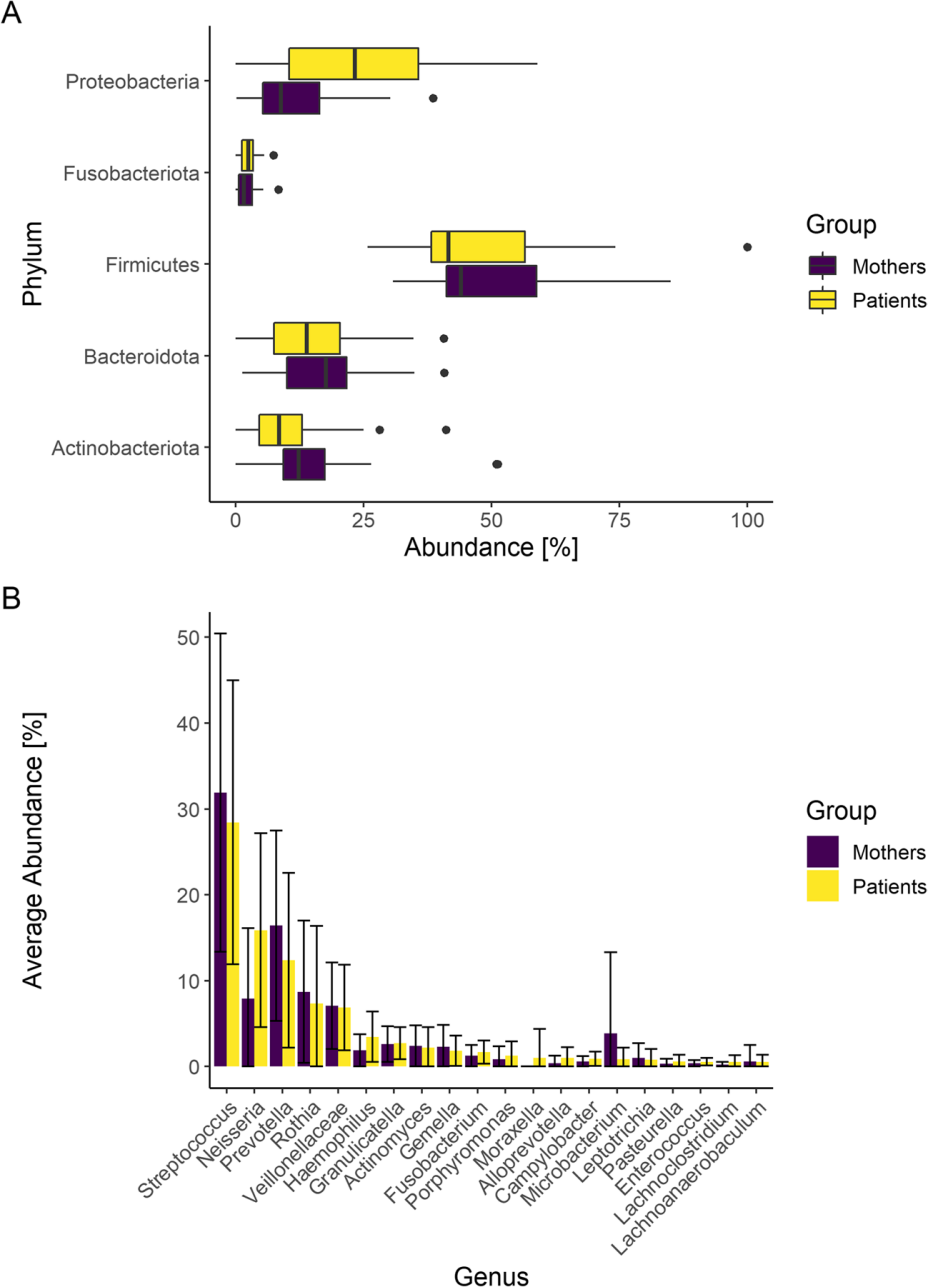
**A**/**B** Differences in the core microbiome of patients with chronic kidney disease (CKD) (*n*=30) and healthy mothers (*n*=21) at the phylum (**A**) and genus (**B**) levels. **A** At the phylum level, *Proteobacteria* were significantly enriched in patients with CKD (FDR=0.037) (see also Online Resource 2). **B** At the genus level, no statistically significant differences were found between patients with CKD and the healthy control group (see also Online Resource 2)

Comparing both groups at the phylum level, the percentage of *Proteobacteria* was significantly higher in the CKD group (23.65 ± 15.5% vs. 12.1% ± 10.5%, FDR=0.037) (Fig. 1A and Online Resource 2).

Next, samples were analyzed at the genus level. Without further postprocessing, we found 210 genera in our data set, of which 61 genera (29% of all genera) were found exclusively in the control group. These 61 genera were present in very small quantities collectively accounting for only 0.02% of the control group (Online Resource 3); hence, the impact on genus-level diversity was small, and accordingly diversities did not vary between the patients and controls (*p* > 0.05). All the genera with a mean relative abundance of at least 0.5% in the samples (*n*=20 core genera) were considered separately and compared between the two groups (Fig. 1B); these 20 most abundant genera accounted for 90.8% of the study group and 90.7% of the control group. In the group of children with CKD, *Streptococcus* spp. (28.4% ± 16.5%) were the most abundant genera followed by *Neisseria* spp. (15.9% ± 11.3%) and *Prevotella* spp. (12.4% ± 10.2%). No statistically significant difference was found in the relative abundance of genera between the two groups (corrected FDRs>0.05, Online Resource 3).

We found 282 species in our dataset. Statistically, there was no difference in the relative abundance of species between patients and controls (FDR>0.05 for all species) in the raw analysis (see Supplementary Table 3).

#### Alpha diversity and richness at species and genus level

**Fig. 2:**
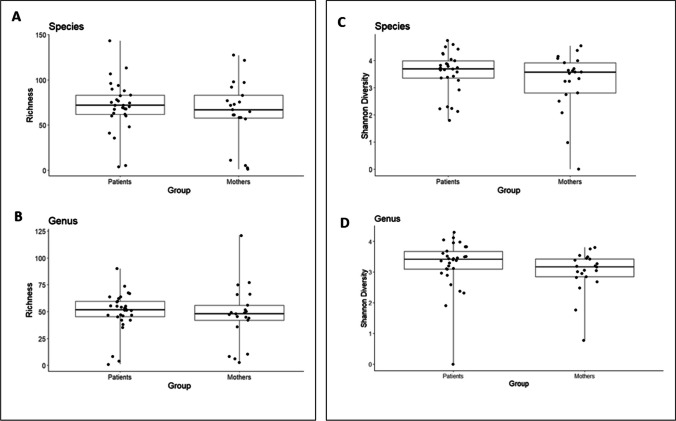
Comparison of richness (**A**/**B**) and alpha diversity (Shannon diversity Index) (**C**/**D**) at genus and at species level

**Fig. 3:**
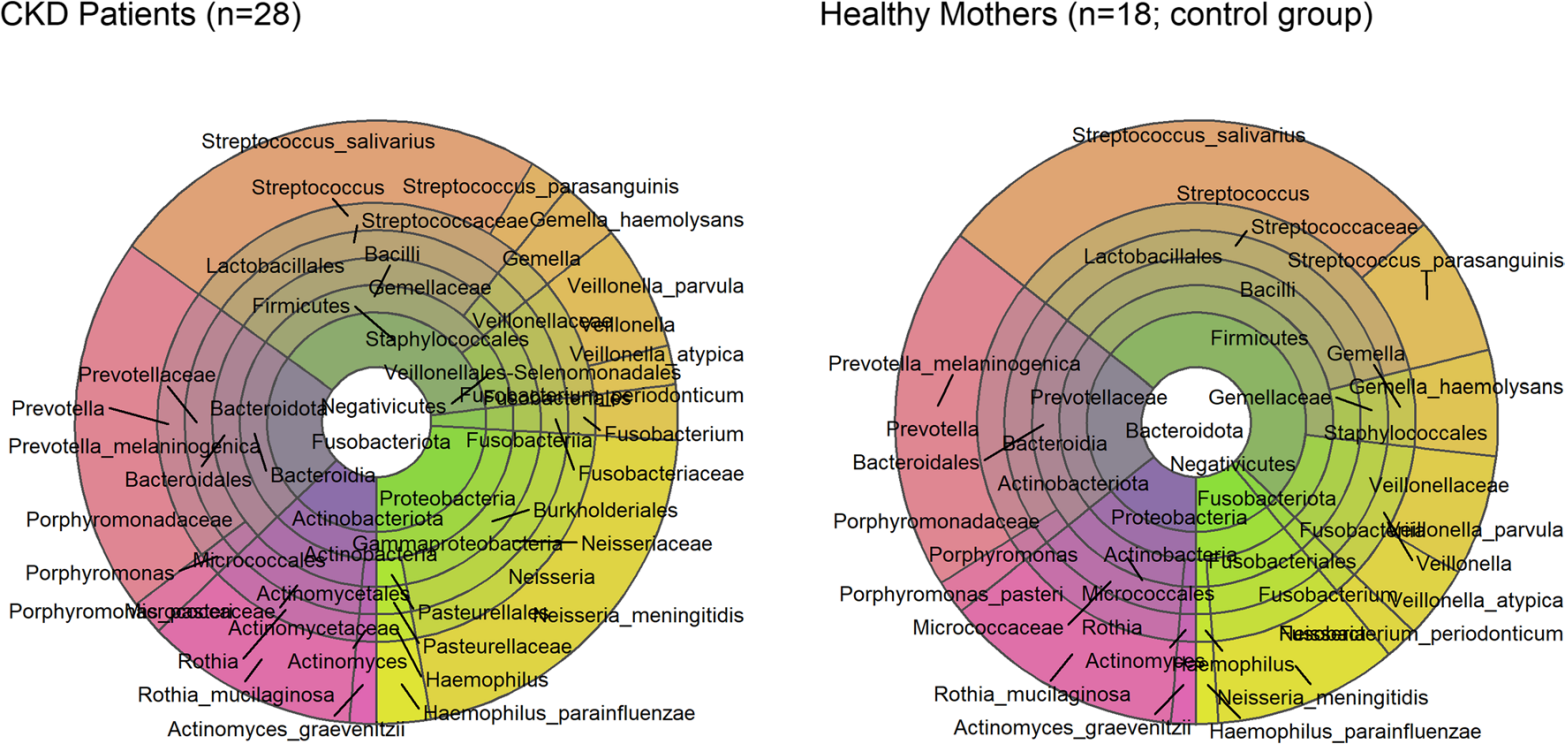
The Krona graph showing the relative abundance of patients with chronic kidney disease (CKD) and healthy mothers (control group)

There was no difference in the richness between the two groups at genus level (mothers 47.38 ± 27.09 vs. patients 49.57 ± 18.9, *p*=0.75) and at species level (mothers 64.86 ± 35.63 vs. patients 71.13 ± 28.25, *p*=0.51) (Fig. 2A/B). The alpha diversity of the studied patients in comparison to the maternal control group also appears similar (estimated by the Shannon Index—compare Fig. 3, two-sided two-sample *t*-test *p*-value=0.2521/0.2747 (species level/genus level) (Fig. 2C/D)).

For further and more specific analysis, we had a closer look at the core microbiome (low abundant (f<2.5%) and rare species (occurrence in less than 10 samples) were excluded from analysis)

#### Analysis of the core OTUs at a species level

In the patients and healthy mothers, we identified 12 core OTUs at the species level. These accounted for an average of 38.8% (± 11%) of the reads per sample (compared with the OTUs that were assigned at the phylum/genus level). Mean relative abundances per group of taxa down to the species level are visualized as a Krona graph in Fig. 3.

*Streptococcus salivarius* (patients with CKD, 23.72 ± 19.99; healthy mothers, 27.98 ± 20.5), *Neisseria meningitidis* (21.17 ± 16.5; 9.87 ± 9.48), and *Prevotella melaninogenica* (20.22 ± 13.86; 19.55 ± 15.16) were the three most abundant species. When comparing the relative abundance of the different species, we found that the relative abundance of *Neisseria meningitidis* was significantly increased in the patients with CKD (21.17% ± 16.5 vs. 9.87% ± 9.48, FDR=0.0308), whereas the relative abundance of *S. parasanguinis* was significantly decreased (2.24% ± 2.77 vs. 7.54 ± 5.27, FDR=0.008) (Table 2).

**Table 2:** Relative abundance at species level

Phylum	Class	Order	Family	Genus	Species	CKD patients		Healthy mothers (control group)		*p*-value	FDR
						Mean %	SD %	Mean %	SD %		
Firmicutes	Bacilli	Lactobacillales	Streptococcaceae	*Streptococcus*	*Streptococcus_salivarius*	23.72	19.99	27.98	20.50	0.4918	0.8431
Proteobacteria	Gammaproteobacteria	Burkholderiales	Neisseriaceae	*Neisseria*	*Neisseria_meningitidis*	21.17	16.50	9.87	9.48	**0.0051**	**0.0308**
Proteobacteria	Bacteroidia	Bacteroidales	Prevotellaceae	*Prevotella*	*Prevotella_melaninogenica*	20.22	13.86	19.55	15.16	0.8810	0.9954
Fusobacteriota	Actinobacteria	Micrococcales	Micrococcaceae	*Rothia*	*Rothia_mucilaginosa*	11.43	16.50	12.89	13.73	0.7480	0.9954
Firmicutes	Negativicutes	Veillonellales-Selenomonadales	Veillonellaceae	*Veillonella*	*Veillonella_parvula*	6.71	5.36	6.47	7.00	0.9008	0.9954
Firmicutes	Bacilli	Staphylococcales	Gemellaceae	*Gemella*	*Gemella_haemolysans*	3.30	4.68	5.65	7.63	0.2513	0.5026
Firmicutes	Fusobacteriia	Fusobacteriales	Fusobacteriaceae	*Fusobacterium*	*Fusobacterium_periodonticum*	2.96	3.90	1.77	2.75	0.2314	0.5026
Actinobacteriota	Gammaproteobacteria	Pasteurellales	Pasteurellaceae	*Haemophilus*	*Haemophilus_parainfluenzae*	2.89	3.89	1.17	2.02	0.0573	0.2292
Bacteroidota	Bacilli	Lactobacillales	Streptococcaceae	*Streptococcus*	*Streptococcus_parasanguinis*	2.24	2.77	7.54	5.27	**0.0007**	**0.0080**
Firmicutes	Negativicutes	Veillonellales-Selenomonadales	Veillonellaceae	*Veillonella*	*Veillonella_atypica*	2.08	3.98	3.92	5.61	0.2370	0.5026
Actinobacteriota	Bacteroidia	Bacteroidales	Porphyromonadaceae	*Porphyromonas*	*Porphyromonas_pasteri*	1.83	3.94	1.84	4.22	0.9954	0.9954
Bacteroidota	Actinobacteria	Actinomycetales	Actinomycetaceae	*Actinomyces*	*Actinomyces_graevenitzii*	1.44	3.55	1.35	3.70	0.9378	0.9954

Significant values have been highlighted in bold

**Fig. 4:**
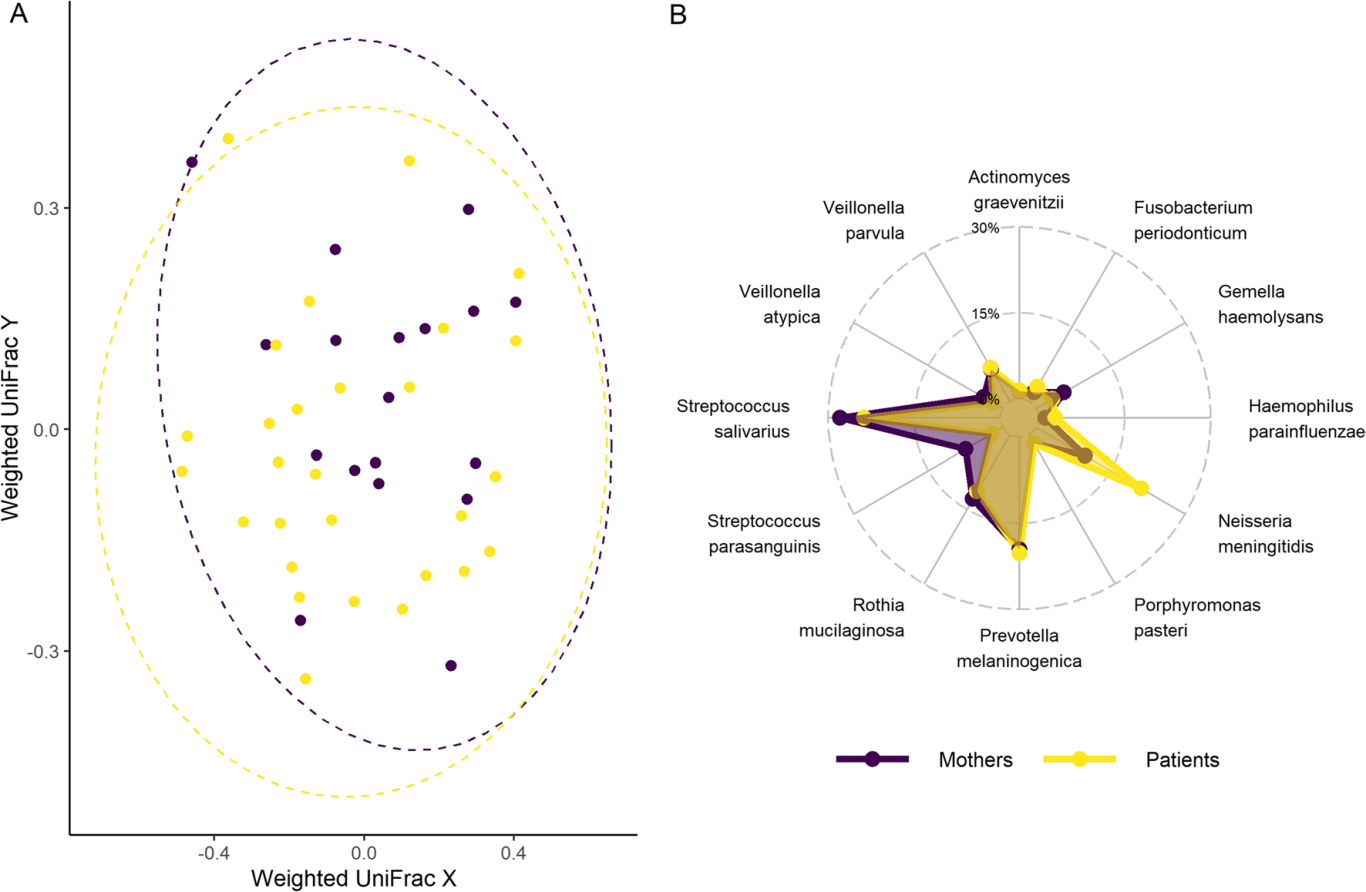
**A**/**B** Beta diversities (**A**) and mean relative abundances (**B**) of the core species. **A** Two-dimensional visualization of the beta diversities. Weighted UniFrac distances were determined on relative abundances of the core species. The degree of overlap between the groups/point clouds represents the similarity of the microbiome profiles between the groups. **B** The radar plot shows average relative abundances [%] of core species in the study groups; the degree of overlap of the covered areas represents the similarity of the core species detected in the groups

For a more conclusive insight, we examined the beta diversity of the study patients and their mothers (Fig. 4A). This revealed that the inter-individual variation was greater than the differences between healthy individuals and those with CKD. Likewise, visualization of mean relative abundances as a radar chart (Fig. 4B) revealed a high degree of overlap in the covered areas, emphasizing the similarity of the microbial communities in our study groups.

#### Impact of the patients’ age on microbial community composition

**Fig. 5:**
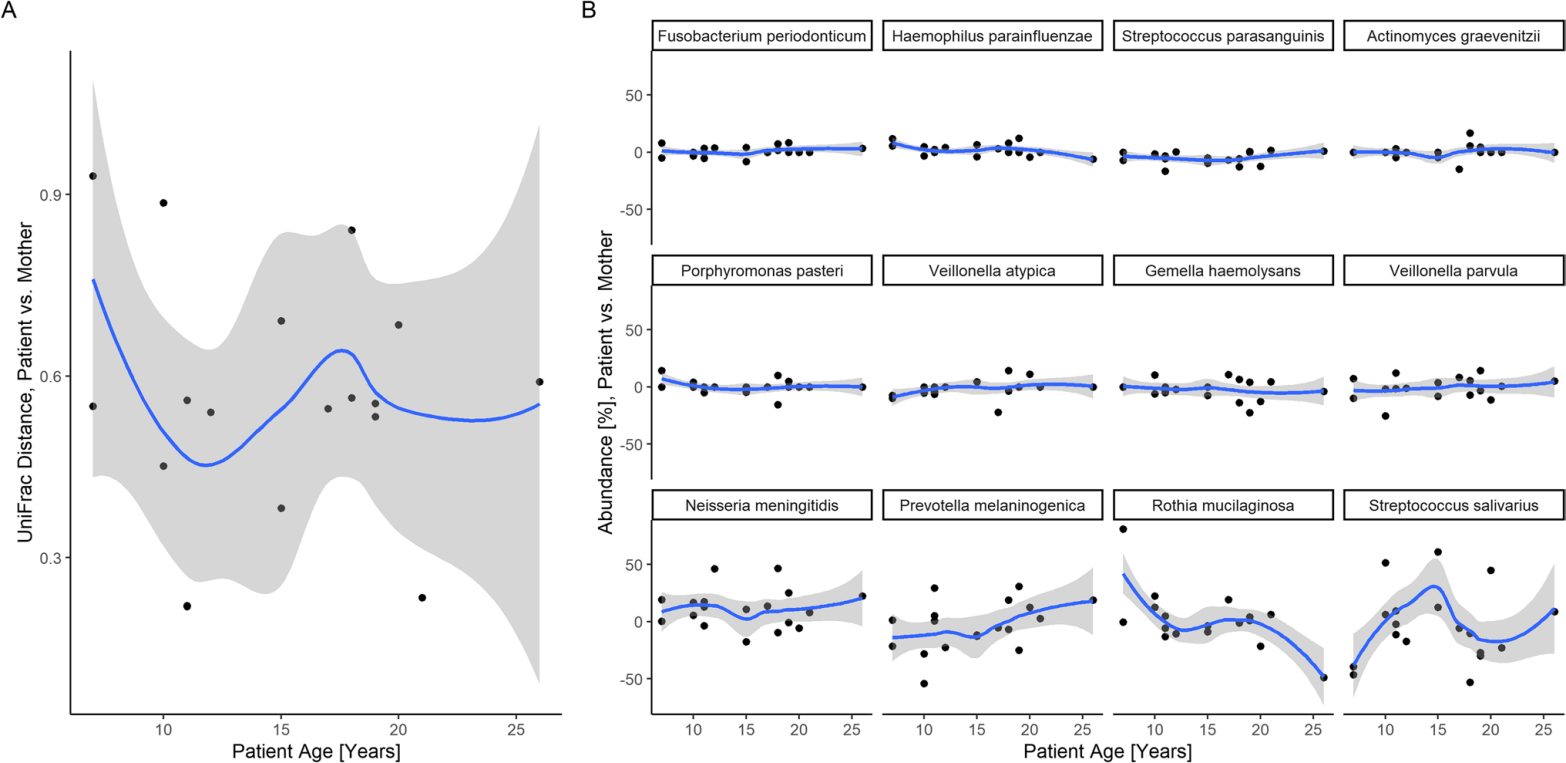
**A**/**B** Visualization of the differences in microbial composition and bacterial relative abundance between patients with chronic kidney disease (CKD) and their mothers by patients’ age. **A** UniFrac distances from patients with CKD to their respective mothers. No clear non-horizontal line outside the confidence interval can be detected, indicating that the UniFrac distances are independent of age. **B** Differences in the bacterial relative abundance between patients with CKD and their mothers according to age

Furthermore, we analyzed whether the age of the patients with CKD has an impact on the microbial community composition; we investigated beta diversities between patients and their mothers and focused on their changes with patient age. Although not statistically significant, trends in certain age ranges prompted further investigation (Fig. 5A).

In the second step, we analyzed the relative abundance differences of each core species between patients and their mothers for changes with patient age (Fig. 5B). We observed that the relative abundance of *Fusobacterium periodonticum*, *Haemophilus parainfluenzae*, *S. parasanguinis*, *Actinomyces graevenitzi*, *Porphyromonas pasteri*, *Veillonella atypica*, *Gemella haemolysans*, and *Veillonella parvula* remained stable with age.

The within-family differences in the relative abundance of *Neisseria meningitidis*, *Prevotella melaninogenica*, *Rothia mucilaginosa*, and *Streptococcus salivarius* showed greater variability with age than the other detected bacterial species.

## Discussion

The present study aimed to investigate whether the tongue microbiomes of children, adolescents, and young adults show measurable alterations compared with a control group. Among the present study participants, the composition of the microbiome at the phylum and genus levels was similar to that of their healthy mothers. Nevertheless, the relative abundance of proteobacteria was significantly higher in the study group. However, no differences in the alpha diversity of the tongue microbiome were observed compared with their mothers. Differences in the relative abundance of core OTUs at the species level between relative pairs were found in species with documented dietary dependence (i.e., streptococci) and in species that are known to be age-related rather than disease-related (*N. meningitidis*). Moreover, the distance between the compositions of microbial communities in young patients and their mothers was independent of the patient’s age.

The tongue microbiome represents a consistent and conserved microbiome within the oral cavity [6] and constitutes a large surface area containing a high biofilm biomass that is subjected to bacterial shedding and cellular desquamation [22].

We used healthy mothers from the same household as controls, as it is known, that strain-sharing of the oral microbiome is affected more by cohabitation than by age or genetics. In particular, the mother-to-infant microbiome transmission is considerable and stable during infancy and even remain detectable at older ages [18].

In 2017, Hall et al. analyzed the core oral microbiome (dental, tongue, and salivary samples) of ten healthy patients, including all the OTUs that were present in ≥95% of all the collected samples [23] and found five predominant phyla: *Actinobacteria*, *Bacteroidetes*, *Firmicutes*, *Fusobacterium*, and *Proteobacteria*. This was identical to our data, since approximately 98% of all OTUs in our study group as well as in our control group belonged to these five phyla. Even in the oral microbiome of a Chinese patients with CKD and in their healthy controls described by Guo et al., *Actinobacteria*, *Bacteroidetes*, *Firmicutes*, *Fusobacterium*, and *Proteobacteria* were the most predominant phyla in the oral microbiome (Guo, 2022), as well as in healthy children, suggesting high phylum-level cognition [24, 25].

Even at the genus level, the core microbiome seems to be very consistent, and the core genera described by Hall et al. (*Streptococcus*, *Fusobacterium*, *Haemophilus*, *Neisseria*, *Prevotella*, and *Rothia*) were also found to be predominant in our dataset (Fig. 1B); however, we did not find any statistically significant differences in the relative abundance of the genera when comparing young patients with CKD and their healthy mothers. Thus, the present data confirm a high similarity between young patients with CKD and healthy individuals. A direct comparison of our patient population with a historical control group of healthy children [26] shows high similarity at the genus level (see Supplementary Table 4). Differences at the species level cannot be meaningfully evaluated because different databases were used in the different trials. Despite this limitation, we have not identified differences between the tongue microbiome of young CKD patients and that of young healthy children at species level. No changes were observed within the disease period

We have refrained from discussing the core microbiome at the species level because the OTU classification at the species level is highly dependent on the bioinformatic pipeline, especially on the database used for OTU assignment, which differ between our study and the mentioned studies.

According to the literature, both exogenous and endogenous factors influence the human microbiome. Thus, an imbalanced human microbiome is associated with CKD, not only due to CKD-associated factors, such as uremia, increased inflammation, and immunosuppression, but also due to pharmacological therapies and dietary restrictions [3]. These influences on imbalance have been predominantly described in adult patients with CKD and their gut microbiome [3]. Limited data are available on the oral microbiome of children, adolescents, and young adults with CKD. Moreover, a comparable study on the relationship between CKD and the oral microbiome in adult patients was conducted using pharyngeal swabs [27]. According to Guo et al., the microbial diversity of patients with CKD is higher than that of healthy controls. Thus, potential oral microbial markers have been identified as non-invasive tools for CKD diagnosis [27]. In contrast, no impact of CKD on the imbalance in the tongue microbiome could be detected in the young patients with CKD. In principle, changes in the microbiome of patients with CKD seem to correlate with the duration of dialysis [3]. In this study population, the mean duration of CKD was 11 years but only two of the 30 patients underwent dialysis (with a maximum of 6 years). Despite the relevant duration of disease, a deviation of the tongue microbiome in comparison to that of their mothers could not be detected. Therefore, the duration of dialysis seems to be of more importance.

In general, our study group seems to be clinically representative, since low caries prevalence and generalized gingivitis were consistent with that of other reports [28]. Several hypotheses exist regarding how an increased incidence of gingivitis could arise in CKD. Besides an altered tissue response as a result of immunosuppression and uremia, an inflammatory response to plaque and calculus accumulation was more frequently reported in studies of patients with CKD [29]. Limited research has been conducted on whether an altered microbiota composition results in an increased prevalence of gingivitis.

The tongue microbiome could be the reservoir for gingivitis flora and that the number of gingivitis species in the tongue microbiome is lower in healthy patients than in patients with periodontitis and gingivitis [30].

Overall, five of the 30 typical (most abundant) gingivitis species germs (Abusleme et al., 2021) were detected in the tongue microbiome of our study group: *Fusobacterium periodonticum*, *Prevotella melaninogenica*, *Veillonella parvula*, *Porphyromonas pasteri*, *Haemophilus parainfluenzae*. Compared to healthy individuals, gingivitis communities are enriched primarily with gram-negative anaerobic species, although oxygen consumers, such as *Neisseria* spp. and *Streptococcus* spp., are also among the enriched taxa [30].

Community alpha diversity, which is a measure of the number of species (richness) and their distribution (evenness), did not differ between healthy individuals and those with periodontitis; however, it was higher in gingivitis [30]. In contrast to the tongue microbiome, the subgingival microbiome revealed that both richness and diversity increase during gingivitis, while richness remains high during periodontitis because no species are lost during the shift, some species appear to become dominant (i.e., their proportion increases) in the periodontitis-associated communities, thereby increasing the uniformity of the community and decreasing the overall diversity compared to gingivitis. In our study, no differences in the alpha diversity or richness were identified between the study and control groups. Our patient sample is representative of patients with CKD, as gingivitis has also been described by other authors [27]. However, in comparison to the mothers, we noticed that *N. meningitidis* and *S. salivarius*/*parasanguinis* were different. *N. meningitidis* was detected more frequently in young study patients than in mothers, whereas *S. parasanguinis* was detected more frequently in mothers.

We assume that this effect was not due to disease versus health, but possibly indicates an age effect of the oral microbiome probably. According to current studies, prevalence of *N. meningitidis* (assessed by cultural methods) increased through childhood from 4.5% in infants to a peak of 23.7% in 19-year-olds and subsequently decreased in adulthood to 7.8% in 50-year-olds [31]. This is consistent with our data, which showed significantly higher levels of *N. meningitidis* in the sample of young patients with CKD (mean age, 14; *N. meningitidis*, 21.2%) compared to healthy mothers (mean age, 41; *N. meningitidis* 9.9%).

Recent studies on the microbial ecology of the oral cavity have demonstrated that *S. parasanguinis*, *S. infantis*, *S. australis*, *S. rubneri*, and *S. salivarius* are among the specialists of the tongue dorsum, and *S. parasanguinis*, *Streptococcus australis*, and *S. salivarius* are the most abundant species on the dorsum of the tongue [32]. *S. parasanguinis* is a commensal gram-positive bacterium, which is considered a primary colonizer of the human oral cavity and is involved in the formation of dental plaque [33]. In addition, it appears to be an antagonist of periodontal pathogens but has the lowest inhibitory potential of all inhibitory species [34]. Previous studies have reported that sucrose phases, in particular, are characterized by a significant increase in the relative abundance of streptococci, including *S. parasanguinis*. Consequently, the higher frequency of *S. parasanguinis* in the mothers could be explained by a higher carbohydrate consumption compared to the diet-controlled nutrition of patients with CKD. Interestingly, patients with CKD had nearly no carious lesions, which seems to support this hypothesis.

### Limitations

The originally intended control of healthy siblings could not be included and should be planned for a follow-up study. The mothers did not undergo a clinical examination; therefore, no information regarding the local inflammation (PBI, QHI) in the oral cavity could be provided. The information on oral health was recorded on the medical history of the mothers. We assumed that the mothers had a normal gingivitis prevalence comparable to that of historical controls [35]. The study included a heterogeneous age of the study patients (6–25 years), no fecal samples were obtained to compare the intestinal microbiome, and a small number of patients were included to generalize or perform a subgroup analysis. This study could be underpowered and should be evaluated in the context of rare diseases [36]. These limitations should be considered for a more detailed plan and hypothesis generation for future studies.

## Conclusions

For young patients with CKD and generalized gingivitis, the following conclusions were drawn with respect to the tongue microbiome. (1) No differences in alpha diversity of the tongue microbiome compared to their mothers were observed (*p* > 0.05). The composition of the core microbiome was similar to that of healthy mothers and historically healthy controls. (2) The distance between the composition of microbial communities in young patients and their mothers is independent of the patient’s age. Differences in relative abundance between relative pairs were found in species with documented dietary restrictions and age-dependence. (3) With a mean disease duration of 11 years and a maximum of 22 years, no significant differences were observed. Duration of dialysis might be responsible for alterations in the microbiome. In conclusion, CKD and its metabolic changes have no detectable impact on the stable tongue microbiome observed in young patients. Compared to documented historical controls, young CKD patients do not present dramatic changes in the tongue microbiome (at species level).

### Supplementary information

ESM 1

## Data Availability

Sequencing data and sample metadata are available at the Sequence Read Archive (https://www.ncbi.nlm.nih.gov/sra) under accession number PRJNA938485.
