# Supplementary material for: The tongue microbiome of young patients with chronic kidney disease and their healthy mothers
Source: Clin Oral Investig. 2024 Jan 24;28(1):110. doi: 10.1007/s00784-024-05492-x (PMC10808353; doi:10.1007/s00784-024-05492-x)
Supplement: Supplementary file 1 — ESM 1 [file 784_2024_5492_MOESM1_ESM.pdf]

Clinical Oral Investigations: Supplementary Material

The tongue microbiome of young patients with chronic kidney disease and their healthy mothers

Karolin C. Höfer[1*], Lutz T. Weber[2], Greta Barbe[1], Isabelle Graf[3], Stefanie Thom[4], Angela Nowag[4], Claus J. Scholz[4], Hilmar Wisplinghoff[4,6], Michael J. Noack[1], Nathalie Jazmati[4,5]

*** Corresponding author:**

Dr. Karolin C. Höfer,

University of Cologne, Polyclinic for Operative Dentistry and Periodontology

Kerpener Str. 32, 50931 Cologne, Germany

Phone: 0049 221 478 96743

Fax number: 0049 221 478 96755

E-mail: karolin.hoefer@uk-koeln.de

ORCID ID: 0000-0001-8284-9849

Online Resources

This supplementary material is hosted by the **Journal Clinical Oral Investigantions** as supporting information alongside the article “**The tongue microbiome of young patients with chronic kidney disease and their healthy mothers”** on behalf of the authors, who remain responsible for the accuracy and appropriateness of the content. The same standards for ethics, copyright, attributions and permissions as for the article apply.

Online Resource 1: Rarefraction analysis for species level analysis

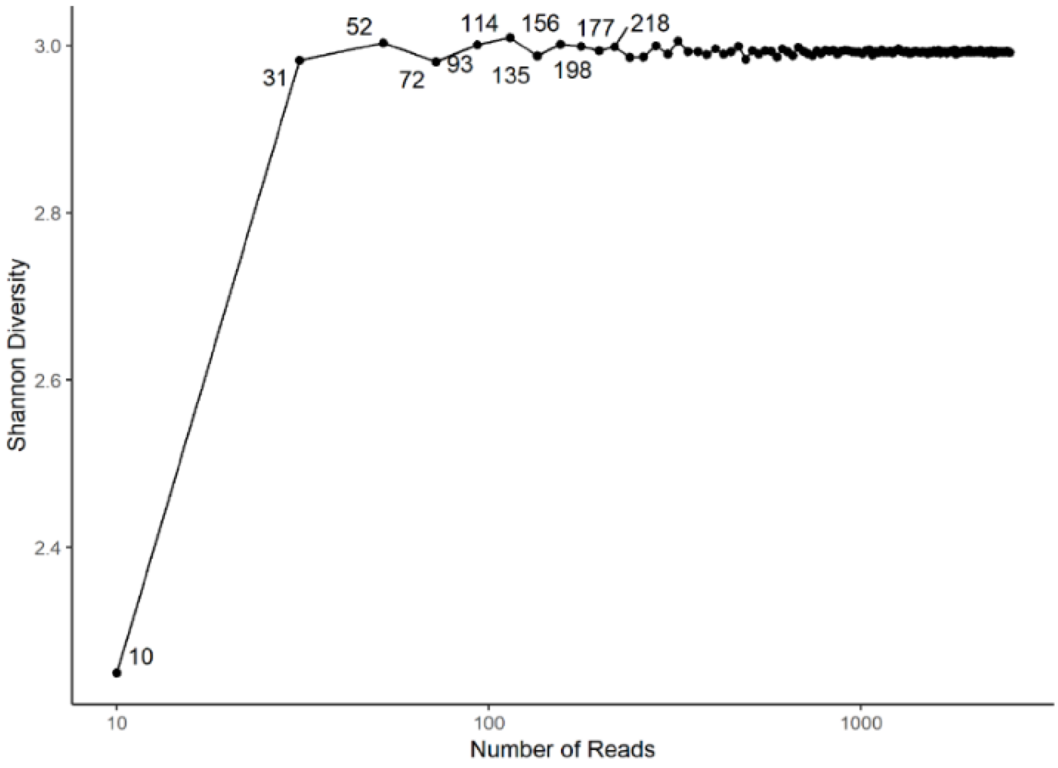

Supplementary table 1: Relative abundance at phylum level

	CKD patients		healthy mothers (control group)		p-value	FDR	core_FDR
Phylum	mean %	sd %	mean %	sd %			
Firmicutes	46.247860	15.988630	50.210271	14.647262	0.364820	0.521536	1.000000
Proteobacteria	23.647833	15.510258	12.106690	10.516507	**0.002656**	**0.037183**	**0.013280**
Bacteroidota	15.084620	10.191141	17.803471	10.876416	0.372526	0.521536	1.000000
Actinobacteriota	10.954113	8.944728	16.001057	13.416567	0.141941	0.521536	0.567764
Fusobacteriota	2.485070	1.866143	2.276705	2.214216	0.726448	0.726448	1.000000
Campilobacterota	0.894623	0.819168	0.600381	0.626991	0.153043	0.521536	NA
Patescibacteria	0.676200	0.746077	0.988743	0.954531	0.217236	0.521536	NA
Cyanobacteria	0.006077	0.020275	0.008886	0.020441	0.630408	0.678901	NA
Spirochaetota	0.003037	0.006699	0.002124	0.004460	0.561906	0.655557	NA
Synergistota	0.000523	0.001933	0.001514	0.003734	0.274025	0.521536	NA
Desulfobacterota	0.000033	0.000183	0.000086	0.000271	0.445554	0.567069	NA
Myxococcota	0.000013	0.000073	0.000000	0.000000	0.325582	0.521536	NA
Bdellovibrionota	0.000000	0.000000	0.000010	0.000044	0.329257	0.521536	NA
Verrucomicrobiota	0.000000	0.000000	0.000081	0.000371	0.329257	0.521536	NA

Supplementary table 2: Relative abundance at genus level

	CKD patients		healthy mothers (control group)		p-value	FDR	core_FDR
Genus	mean %	sd %	mean %	sd %			
Streptococcus	28.442547	16.543809	31.883014	18.533093	0.499429	0.620592	1.000000
Neisseria	15.865013	11.302233	7.925243	8.175825	**0.005416**	0.493885	0.108322
Prevotella	12.384470	10.189428	16.402224	11.098937	0.195666	0.493885	1.000000
Rothia	7.322763	9.037988	8.701648	8.280824	0.575875	0.683242	1.000000
Veillonellaceae	6.875607	4.974429	7.083400	5.034429	0.884779	0.899222	1.000000
Haemophilus	3.464123	2.953565	1.878171	1.890429	**0.023658**	0.493885	0.449510
Granulicatella	2.710783	1.856053	2.621348	2.062683	0.874669	0.899222	1.000000
Actinomyces	2.218410	2.362723	2.384510	2.427366	0.809063	0.853785	1.000000
Gemella	1.854873	1.767279	2.291067	2.574191	0.505435	0.624361	1.000000
Fusobacterium	1.665900	1.354008	1.260295	1.255878	0.277604	0.493885	1.000000
Porphyromonas	1.255840	1.680330	0.844024	1.512985	0.365630	0.510700	1.000000
Moraxella	1.009257	3.384791	0.003676	0.011337	0.114512	0.493885	1.000000
Alloprevotella	0.980367	1.244983	0.402452	0.869561	0.056692	0.493885	0.963760
Campylobacter	0.894547	0.819076	0.600138	0.627093	0.152821	0.493885	1.000000
Microbacterium	0.840273	1.340528	3.866505	9.450591	0.160166	0.493885	1.000000
Leptotrichia	0.818367	1.215076	1.016386	1.682206	0.647243	0.729783	1.000000
Pasteurella	0.572793	0.788168	0.348310	0.572464	0.244485	0.493885	1.000000
Enterococcus	0.559360	0.418146	0.385290	0.355274	0.116330	0.493885	1.000000
Lachnoclostridium	0.557210	0.731125	0.238152	0.280565	**0.035805**	0.493885	0.644497
Lachnoanaerobaculum	0.530877	0.849796	0.597938	1.890943	0.880279	0.899222	1.000000
Peptostreptococcaceae	0.455550	1.153334	0.408090	1.165383	0.886376	0.899222	NA
Capnocytophaga	0.421220	0.731407	0.136071	0.286112	0.060113	0.493885	NA
Oribacterium	0.396983	0.751712	0.249490	0.398218	0.368606	0.510700	NA
Lactobacillus	0.334533	0.285686	0.395319	0.388942	0.545737	0.654885	NA
Saccharimonadaceae_TM7x	0.329827	0.482259	0.557190	0.636790	0.175610	0.493885	NA
Serratia	0.322407	0.491028	0.193767	0.340440	0.274612	0.493885	NA
UCG 14	0.322387	0.493788	0.142710	0.211955	0.083427	0.493885	NA
Solobacterium	0.264917	0.350750	0.224710	0.295646	0.660280	0.729783	NA
Veillonellaceae	0.261390	0.498347	0.530724	0.646216	0.117296	0.493885	NA
Atopobium	0.217883	0.328266	0.264895	0.357226	0.635126	0.729783	NA
Saccharimonadales	0.181223	0.288055	0.264567	0.351789	0.376156	0.512940	NA
Acinetobacter	0.181077	0.348150	0.113976	0.259462	0.434354	0.560714	NA
Veillonellaceae	0.175473	0.853846	0.023324	0.047172	0.338100	0.496614	NA
Catonella	0.160323	0.563498	0.027514	0.033363	0.207935	0.493885	NA
Butyrivibrio	0.147863	0.492003	0.063748	0.113142	0.373052	0.512033	NA
Selenomonadaceae	0.123413	0.199887	0.297290	0.642246	0.242262	0.493885	NA
Stomatobaculum	0.115917	0.186597	0.180648	0.223924	0.284029	0.493885	NA
Lautropia	0.098417	0.259821	0.024948	0.053585	0.142309	0.493885	NA
Anaerovoracaceae	0.089167	0.143339	0.939876	3.830324	0.321144	0.493885	NA
Rodentibacter	0.085283	0.192018	0.006524	0.023898	**0.033890**	0.493885	NA
Aggregatibacter	0.078743	0.113744	0.024395	0.048475	**0.024582**	0.493885	NA
Actinobacillus	0.075360	0.128902	0.107352	0.456606	0.757584	0.820065	NA

Candidatus	0.071517	0.112281	0.110410	0.200581	0.427599	0.558417	NA
Micrococcus	0.066310	0.350462	0.000105	0.000480	0.309364	0.493885	NA
Saccharimonadaceae	0.044633	0.064669	0.041500	0.082388	0.884982	0.899222	NA
Corynebacterium	0.043700	0.077325	0.134333	0.581247	0.485600	0.607000	NA
Absconditabacteriales	0.035957	0.077903	0.009505	0.016819	0.080941	0.493885	NA
Carnobacterium	0.030953	0.046241	0.014938	0.043913	0.216372	0.493885	NA
Anaerovoracaceae	0.029753	0.036550	0.054038	0.148155	0.469821	0.594352	NA
Peptostreptococcaceae	0.024457	0.083456	0.007605	0.020010	0.295270	0.493885	NA
Mannheimia	0.020463	0.029395	0.029857	0.118471	0.725439	0.793449	NA
Staphylococcus	0.017343	0.093996	0.000924	0.002972	0.346906	0.498545	NA
Trichococcus	0.016100	0.088032	0.002438	0.010210	0.406443	0.540209	NA
Thiobacillus	0.013983	0.046103	0.000162	0.000742	0.111438	0.493885	NA
Tannerella	0.012717	0.032121	0.008062	0.020495	0.530902	0.642541	NA
Bacteroides	0.012370	0.029616	0.000052	0.000240	**0.030279**	0.493885	NA
Saccharimonadaceae_TM7a	0.011563	0.050188	0.004486	0.012358	0.463811	0.590305	NA
Peptostreptococcaceae	0.010290	0.027939	0.003805	0.010133	0.250516	0.493885	NA
Johnsonella	0.010110	0.023548	0.004671	0.010548	0.270971	0.493885	NA
Shewanella	0.008033	0.010819	0.000000	0.000000	**0.000333**	0.070001	NA
Flavobacterium	0.007847	0.021645	0.006076	0.021029	0.771395	0.826494	NA
Cardiobacterium	0.007413	0.009603	0.006467	0.014883	0.799228	0.847666	NA
Lactococcus	0.006810	0.033349	0.000057	0.000262	0.276529	0.493885	NA
Chloroplast	0.005983	0.020297	0.008852	0.020350	0.622376	0.726105	NA
Brachymonas	0.005857	0.021772	0.000205	0.000617	0.165941	0.493885	NA
Chryseobacterium	0.005677	0.020499	0.000881	0.003902	0.220568	0.493885	NA
Peptostreptococcales	0.005547	0.011290	0.004833	0.019437	0.880802	0.899222	NA
Peptostreptococcales	0.005437	0.010302	0.003971	0.009127	0.595296	0.698392	NA
Simonsiella	0.004607	0.013088	0.000671	0.002370	0.117404	0.493885	NA
Anaerovoracaceae	0.003837	0.020864	0.000252	0.001157	0.355469	0.500996	NA
Acidovorax	0.003817	0.010458	0.000329	0.001171	0.080189	0.493885	NA
Defluviitaleaceae	0.003527	0.008655	0.002129	0.004008	0.443047	0.567316	NA
Arcanobacterium	0.003320	0.018184	0.000086	0.000393	0.338170	0.496614	NA
Abiotrophia	0.003313	0.008050	0.001524	0.004517	0.317061	0.493885	NA
Bifidobacterium	0.003297	0.017720	0.043724	0.193099	0.350062	0.498545	NA
Alkalibacterium	0.003163	0.006492	0.000000	0.000000	**0.012329**	0.493885	NA
Treponema	0.003037	0.006699	0.002124	0.004460	0.561906	0.670456	NA
Globicatella	0.002493	0.006749	0.000371	0.000999	0.100022	0.493885	NA
Kingella	0.002403	0.008458	0.001586	0.003817	0.643563	0.729783	NA
Anaerovoracaceae	0.002140	0.011721	0.002148	0.009030	0.997921	0.997921	NA
Anaerovoracaceae	0.001553	0.007195	0.002586	0.008090	0.641522	0.729783	NA
Actinomycetaceae_F0332	0.001280	0.003657	0.000257	0.000941	0.152230	0.493885	NA
RF39	0.001267	0.004872	0.005114	0.014021	0.239286	0.493885	NA
Lachnobacterium	0.001063	0.005824	0.000195	0.000895	0.428119	0.558417	NA
Alloscardovia	0.001037	0.005584	0.118943	0.361089	0.150205	0.493885	NA
Rikenellaceae	0.000990	0.003335	0.000481	0.001687	0.478016	0.601097	NA
Alysiella	0.000960	0.004577	0.000943	0.004321	0.989204	0.993937	NA
Paludibacteraceae_F0058	0.000753	0.002885	0.002652	0.007082	0.255869	0.493885	NA
Lachnospiraceae	0.000643	0.003524	0.000476	0.002182	0.835461	0.877234	NA
Paenarthrobacter	0.000630	0.002645	0.000000	0.000000	0.202223	0.493885	NA
Bergeyella	0.000613	0.002867	0.000081	0.000371	0.322816	0.493885	NA
Aeromonas	0.000567	0.003104	0.000276	0.001266	0.647383	0.729783	NA
Fretibacterium	0.000523	0.001933	0.001514	0.003734	0.274025	0.493885	NA
Clostridia_vadinBB60_group	0.000433	0.001523	0.000043	0.000196	0.175147	0.493885	NA
Streptobacillus	0.000413	0.001575	0.000000	0.000000	0.161295	0.493885	NA
Shuttleworthia	0.000413	0.002264	0.001176	0.003629	0.399682	0.538033	NA
Peptostreptococcaceae	0.000383	0.001574	0.000000	0.000000	0.192594	0.493885	NA
Mycoplasma	0.000367	0.001356	0.000105	0.000289	0.312737	0.493885	NA
Actinotignum	0.000330	0.001084	0.000000	0.000000	0.106227	0.493885	NA
Moryella	0.000310	0.001698	0.000000	0.000000	0.325582	0.493885	NA
Vibrio	0.000303	0.000715	0.000000	0.000000	**0.027376**	0.493885	NA
Sphingomonas	0.000300	0.001115	0.003386	0.014792	0.351355	0.498545	NA
Scardovia	0.000273	0.001165	0.000000	0.000000	0.209044	0.493885	NA
Burkholderia	0.000247	0.001351	0.000114	0.000524	0.628943	0.729713	NA
Gracilibacteria	0.000227	0.000719	0.000010	0.000044	0.109736	0.493885	NA
Slackia	0.000227	0.001242	0.000114	0.000524	0.660264	0.729783	NA
Pseudomonas	0.000223	0.001060	0.003648	0.015661	0.329008	0.493885	NA
Snodgrassella	0.000207	0.001132	0.000067	0.000306	0.523345	0.642541	NA
Gracilibacteria_69_P22	0.000187	0.000660	0.000124	0.000342	0.659329	0.729783	NA
Oceanivirga	0.000167	0.000913	0.000000	0.000000	0.325582	0.493885	NA
Lactobacillales_p5D1_392	0.000147	0.000803	0.000000	0.000000	0.325582	0.493885	NA
Veillonellaceae	0.000140	0.000767	0.000090	0.000415	0.767713	0.826494	NA
Pelomonas	0.000133	0.000730	0.000410	0.001877	0.527346	0.642541	NA
Enhydrobacter	0.000127	0.000694	0.000133	0.000377	0.964990	0.974268	NA
Eikenella	0.000123	0.000604	0.000048	0.000218	0.532391	0.642541	NA
Howardella	0.000123	0.000676	0.000186	0.000851	0.781195	0.832745	NA
Mobiluncus	0.000120	0.000657	0.000705	0.002964	0.383994	0.520250	NA
Enterobacter	0.000093	0.000413	0.000000	0.000000	0.225363	0.493885	NA

Nostoc	0.000093	0.000511	0.000000	0.000000	0.325582	0.493885	NA
Lentimicrobium	0.000083	0.000438	0.000019	0.000068	0.435221	0.560714	NA
Polynucleobacter	0.000083	0.000456	0.000033	0.000153	0.580768	0.685175	NA
Cryptobacterium	0.000077	0.000420	0.000100	0.000458	0.854011	0.892251	NA
Psychrobacter	0.000073	0.000280	0.002324	0.010649	0.344493	0.498545	NA
Olsenella	0.000070	0.000383	0.000000	0.000000	0.325582	0.493885	NA
Tessaracoccus	0.000060	0.000242	0.000000	0.000000	0.184141	0.493885	NA
Legionella	0.000060	0.000329	0.000000	0.000000	0.325582	0.493885	NA
Comamonas	0.000060	0.000329	0.000110	0.000419	0.653580	0.729783	NA
Buttiauxella	0.000053	0.000292	0.000000	0.000000	0.325582	0.493885	NA
Candidatus	0.000053	0.000218	0.000095	0.000436	0.687904	0.756334	NA
Incertae	0.000047	0.000256	0.000000	0.000000	0.325582	0.493885	NA
Mitochondria	0.000043	0.000237	0.000000	0.000000	0.325582	0.493885	NA
Ilumatobacter	0.000040	0.000219	0.000000	0.000000	0.325582	0.493885	NA
Fluviicola	0.000040	0.000219	0.000000	0.000000	0.325582	0.493885	NA
Clostridium	0.000040	0.000219	0.000290	0.001331	0.402725	0.538677	NA
Desulfovibrio	0.000033	0.000183	0.000000	0.000000	0.325582	0.493885	NA
Novosphingobium	0.000030	0.000164	0.000048	0.000218	0.756087	0.820065	NA
Actinobacteria_PeM15	0.000027	0.000146	0.000000	0.000000	0.325582	0.493885	NA
Peptostreptococcales	0.000027	0.000146	0.000167	0.000764	0.416167	0.549654	NA
Sulfurifustis	0.000020	0.000110	0.000000	0.000000	0.325582	0.493885	NA
Limnohabitans	0.000020	0.000110	0.000000	0.000000	0.325582	0.493885	NA
Sporichthyaceae_hgcI	0.000017	0.000091	0.000000	0.000000	0.325582	0.493885	NA
Paracoccus	0.000017	0.000091	0.002014	0.009231	0.333193	0.496246	NA
Anaerovoracaceae	0.000017	0.000091	0.000210	0.000960	0.369649	0.510700	NA
Paenibacillus	0.000013	0.000073	0.000000	0.000000	0.325582	0.493885	NA
Nannocystis	0.000013	0.000073	0.000000	0.000000	0.325582	0.493885	NA
Rhodovastum	0.000013	0.000073	0.000000	0.000000	0.325582	0.493885	NA
Pseudopropionibacterium	0.000007	0.000037	0.000000	0.000000	0.325582	0.493885	NA
Agreia	0.000003	0.000018	0.000000	0.000000	0.325582	0.493885	NA
Caulobacter	0.000003	0.000018	0.000076	0.000349	0.350770	0.498545	NA
Ochrobactrum	0.000000	0.000000	0.000352	0.000923	0.095474	0.493885	NA
Ensifer	0.000000	0.000000	0.002633	0.007084	0.103947	0.493885	NA
Brevundimonas	0.000000	0.000000	0.000967	0.002921	0.145009	0.493885	NA
Desulfobulbus	0.000000	0.000000	0.000086	0.000271	0.162298	0.493885	NA
Cutibacterium	0.000000	0.000000	0.000133	0.000426	0.166781	0.493885	NA
Methylobacterium	0.000000	0.000000	0.001462	0.004672	0.167016	0.493885	NA
Achromobacter	0.000000	0.000000	0.000610	0.001956	0.168770	0.493885	NA
Parascardovia	0.000000	0.000000	0.000071	0.000231	0.171036	0.493885	NA
Delftia	0.000000	0.000000	0.000481	0.001570	0.175602	0.493885	NA
Rhodococcus	0.000000	0.000000	0.001386	0.004558	0.178845	0.493885	NA
Cellvibrio	0.000000	0.000000	0.000510	0.002097	0.278634	0.493885	NA
Nocardioides	0.000000	0.000000	0.000252	0.001134	0.319898	0.493885	NA
Peptostreptococcaceae	0.000000	0.000000	0.000224	0.001026	0.329257	0.493885	NA
Hydrogenophaga	0.000000	0.000000	0.000214	0.000982	0.329257	0.493885	NA
Georgenia	0.000000	0.000000	0.001943	0.008903	0.329257	0.493885	NA
Mycetocola	0.000000	0.000000	0.000219	0.001004	0.329257	0.493885	NA
Kocuria	0.000000	0.000000	0.000252	0.001157	0.329257	0.493885	NA
Paeniglutamicibacter	0.000000	0.000000	0.000081	0.000371	0.329257	0.493885	NA
Actinoplanes	0.000000	0.000000	0.000348	0.001593	0.329257	0.493885	NA
Mucilaginibacter	0.000000	0.000000	0.000062	0.000284	0.329257	0.493885	NA
Pedobacter	0.000000	0.000000	0.000190	0.000873	0.329257	0.493885	NA
Oligoflexus	0.000000	0.000000	0.000010	0.000044	0.329257	0.493885	NA
Arcobacter	0.000000	0.000000	0.000029	0.000131	0.329257	0.493885	NA
Pseudarcobacter	0.000000	0.000000	0.000052	0.000240	0.329257	0.493885	NA
Tychonema	0.000000	0.000000	0.000033	0.000153	0.329257	0.493885	NA
Anoxybacillus	0.000000	0.000000	0.000086	0.000393	0.329257	0.493885	NA
Planomicrobium	0.000000	0.000000	0.000048	0.000218	0.329257	0.493885	NA
Psychrobacillus	0.000000	0.000000	0.000586	0.002684	0.329257	0.493885	NA
Sporosarcina	0.000000	0.000000	0.000276	0.001266	0.329257	0.493885	NA
Bulleidia	0.000000	0.000000	0.000200	0.000917	0.329257	0.493885	NA
Facklamia	0.000000	0.000000	0.000143	0.000655	0.329257	0.493885	NA
Leuconostoc	0.000000	0.000000	0.000276	0.001266	0.329257	0.493885	NA
Jeotgalicoccus	0.000000	0.000000	0.000071	0.000327	0.329257	0.493885	NA
Ruminiclostridium	0.000000	0.000000	0.000181	0.000829	0.329257	0.493885	NA
Proteiniclasticum	0.000000	0.000000	0.000014	0.000065	0.329257	0.493885	NA
Eubacterium	0.000000	0.000000	0.000033	0.000153	0.329257	0.493885	NA
Eubacterium	0.000000	0.000000	0.000014	0.000065	0.329257	0.493885	NA
Dorea	0.000000	0.000000	0.000019	0.000087	0.329257	0.493885	NA
Azospirillum	0.000000	0.000000	0.000090	0.000415	0.329257	0.493885	NA
Bosea	0.000000	0.000000	0.000162	0.000742	0.329257	0.493885	NA
Devosia	0.000000	0.000000	0.000257	0.001178	0.329257	0.493885	NA
Sphingobium	0.000000	0.000000	0.000081	0.000371	0.329257	0.493885	NA
Alcaligenes	0.000000	0.000000	0.000076	0.000349	0.329257	0.493885	NA
Cupriavidus	0.000000	0.000000	0.000095	0.000436	0.329257	0.493885	NA
Ottowia	0.000000	0.000000	0.000033	0.000153	0.329257	0.493885	NA

Polaromonas	0.000000	0.000000	0.001186	0.005434	0.329257	0.493885	NA
Variovorax	0.000000	0.000000	0.000457	0.002095	0.329257	0.493885	NA
Methylotenera	0.000000	0.000000	0.000033	0.000153	0.329257	0.493885	NA
Duganella	0.000000	0.000000	0.000981	0.004495	0.329257	0.493885	NA
Janthinobacterium	0.000000	0.000000	0.000776	0.003557	0.329257	0.493885	NA
Massilia	0.000000	0.000000	0.001410	0.006459	0.329257	0.493885	NA
Rhodocyclaceae_C39	0.000000	0.000000	0.000210	0.000960	0.329257	0.493885	NA
Dechlorosoma	0.000000	0.000000	0.000010	0.000044	0.329257	0.493885	NA
Rickettsiella	0.000000	0.000000	0.000052	0.000240	0.329257	0.493885	NA
Escherichia	0.000000	0.000000	0.000062	0.000284	0.329257	0.493885	NA
Alkanindiges	0.000000	0.000000	0.000095	0.000436	0.329257	0.493885	NA
Photobacterium	0.000000	0.000000	0.001510	0.006918	0.329257	0.493885	NA
Xanthomonas	0.000000	0.000000	0.000071	0.000327	0.329257	0.493885	NA
Lacunisphaera	0.000000	0.000000	0.000029	0.000131	0.329257	0.493885	NA
Pedosphaeraceae_ADurb	0.000000	0.000000	0.000019	0.000087	0.329257	0.493885	NA
Pedosphaeraceae_Ellin516	0.000000	0.000000	0.000033	0.000153	0.329257	0.493885	NA

Supplementary table 3: Relative abundance at species level (no postprocessing)

	CKD patients		healthy mothers (control group)		p-value	FDR
**Species**	**mean %**	**sd %**	**mean %**	**sd %**		
Haemophilus_influenzae	0.037220	0.049822	0.002267	0.006608	0.001	0.126
Neisseria_elongata	0.016397	0.022107	0.001348	0.002943	0.001	0.126
Streptococcus_parasanguinis	1.130590	0.776606	2.612252	1.856762	0.002	0.155
Haemophilus_haemolyticus	0.044867	0.067347	0.003333	0.007527	0.002	0.155
Haemophilus_pittmaniae	0.621480	0.663394	0.218210	0.263688	0.005	0.247
Shewanella_putrefaciens	0.003603	0.006541	0.000000	0.000000	0.005	0.247
Neisseria_meningitidis	7.134403	5.408059	3.616286	3.475141	0.007	0.275
Granulicatella_adiacens	0.009553	0.017915	0.000581	0.002303	0.011	0.365
Aggregatibacter_aphrophilus	0.050540	0.075755	0.010967	0.028556	0.013	0.365
Haemophilus_parahaemolyticus	0.014610	0.028867	0.000619	0.001863	0.013	0.365
Prevotellaceae_bacterium	0.528050	0.834714	0.121076	0.200068	0.015	0.381
Prevotella_loescheii	0.152497	0.279802	0.024562	0.044482	0.020	0.462
Gemella_morbillorum	0.015363	0.022632	0.004343	0.009795	0.022	0.484
SR1_bacterium	0.009137	0.021043	0.000105	0.000480	0.026	0.484
Actinobacillus_pleuropneumoniae	0.016290	0.035375	0.001167	0.003562	0.027	0.484
Lactobacillus_plantarum	0.009100	0.021565	0.000000	0.000000	0.028	0.484
Bacteroidaceae_bacterium	0.012283	0.029649	0.000000	0.000000	0.031	0.484
Streptococcus_infantis	0.030300	0.036822	0.013376	0.017531	0.034	0.484
Mannheimia_haemolytica	0.012367	0.024395	0.002119	0.006358	0.035	0.484
Porphyromonas_catoniae	0.017423	0.038100	0.002162	0.004014	0.038	0.484
Prevotella_shahii	0.051737	0.117645	0.005410	0.012341	0.041	0.484
Veillonella_rogosae	0.070573	0.183813	0.000000	0.000000	0.044	0.484
Prevotella_pallens	0.246143	0.263320	0.487671	0.491020	0.049	0.484
Shewanella_baltica	0.000610	0.001655	0.000000	0.000000	0.053	0.484
Campylobacter_showae	0.000903	0.002218	0.000076	0.000349	0.054	0.484
Haemophilus_parainfluenzae	1.179723	1.189627	0.667086	0.653983	0.054	0.484
Granulicatella_elegans	0.020783	0.027338	0.008548	0.018039	0.060	0.484
Actinomyces_naeslundii	0.047543	0.096821	0.012305	0.019964	0.062	0.484
Corynebacterium_durum	0.018823	0.041871	0.003748	0.008965	0.065	0.484
Hafnia_psychrotolerans	0.000817	0.002357	0.000000	0.000000	0.068	0.484
Capnocytophaga_sputigena	0.114820	0.270403	0.018510	0.058451	0.068	0.484
Leptotrichia_goodfellowii	0.001093	0.004833	0.031076	0.073028	0.075	0.484
Aggregatibacter_segnis	0.019503	0.028229	0.008500	0.014969	0.078	0.484
Acinetobacter_johnsonii	0.000320	0.001247	0.001671	0.003245	0.082	0.484
Clostridiales_bacterium	0.321353	0.492953	0.142271	0.211687	0.084	0.484
Ochrobactrum_intermedium	0.000000	0.000000	0.000271	0.000686	0.085	0.484
Aerococcaceae_bacterium	0.002493	0.006749	0.000371	0.000999	0.100	0.484
Treponema_medium	0.002360	0.005960	0.000462	0.001261	0.100	0.484
Candidatus_Saccharibacteria	0.102557	0.208616	0.217567	0.262359	0.103	0.484
Lactobacillus_sakei	0.002283	0.008771	0.010786	0.021865	0.104	0.484
Gracilibacteria_bacterium	0.000227	0.000719	0.000010	0.000044	0.110	0.484
Simonsiella_muelleri	0.004607	0.013088	0.000671	0.002370	0.117	0.484
Megasphaera_micronuciformis	0.260760	0.497249	0.529181	0.644546	0.118	0.484
Prevotella_aurantiaca	0.110830	0.379994	0.000000	0.000000	0.121	0.484
Lachnospiraceae_bacterium	0.008267	0.021512	0.001867	0.004100	0.122	0.484
Capnocytophaga_gingivalis	0.047393	0.077748	0.018481	0.055347	0.127	0.484
Prevotella_histicola	0.223267	0.425438	0.560343	0.923152	0.131	0.484
Chryseobacterium_reticulitermitis	0.000423	0.001176	0.000076	0.000349	0.136	0.484
Selenomonas_noxia	0.009640	0.022064	0.030024	0.060111	0.151	0.484
Corynebacterium_matruchotii	0.008117	0.021918	0.001881	0.006919	0.153	0.484
Fusobacterium_periodonticum	1.149917	0.992334	0.772124	0.862169	0.155	0.484
Catonella_morbi	0.045310	0.062904	0.025957	0.031895	0.156	0.484

TM7_phylum	0.164733	0.262831	0.320114	0.442492	0.160	0.484
[Haemophilus]_ducreyi	0.004477	0.015659	0.000352	0.001442	0.162	0.484
Selenomonas_infelix	0.005130	0.019386	0.000062	0.000284	0.163	0.484
Porphyromonas_asaccharolytica	0.000067	0.000255	0.000000	0.000000	0.163	0.484
Brachymonas_denitrificans	0.005857	0.021772	0.000205	0.000617	0.166	0.484
Scardovia_wiggsiae	0.000040	0.000154	0.000000	0.000000	0.167	0.484
Prevotella_denticola	0.001880	0.007518	0.010433	0.026730	0.167	0.484
Oribacterium_sinus	0.000000	0.000000	0.002171	0.006979	0.169	0.484
Parascardovia_denticolens	0.000000	0.000000	0.000071	0.000231	0.171	0.484
Prevotella_oryzae	0.000000	0.000000	0.001433	0.004629	0.171	0.484
[Eubacterium]_minutum	0.000000	0.000000	0.001205	0.003925	0.175	0.484
Clostridiales_bacterium	0.000433	0.001523	0.000043	0.000196	0.175	0.484
Delftia_acidovorans	0.000000	0.000000	0.000481	0.001570	0.176	0.484
Massiliprevotella_massiliensis	0.000093	0.000370	0.000000	0.000000	0.178	0.484
Streptococcus_mutans	0.000097	0.000309	0.003790	0.012226	0.182	0.484
Alloscardovia_omnicolens	0.000477	0.002518	0.044952	0.148683	0.186	0.484
Pasteurellaceae_bacterium	0.003103	0.008959	0.000705	0.003230	0.186	0.484
Serratia_fonticola	0.000103	0.000419	0.000000	0.000000	0.187	0.484
Treponema_lecithinolyticum	0.000000	0.000000	0.000105	0.000354	0.190	0.484
Lautropia_mirabilis	0.060640	0.156413	0.020038	0.048473	0.191	0.484
Actinomyces_oris	0.003950	0.010268	0.001310	0.003003	0.192	0.484
Peptoanaerobacter_stomatis	0.000383	0.001574	0.000000	0.000000	0.193	0.484
Synergistales_bacterium	0.000267	0.001131	0.001071	0.002608	0.196	0.484
Parvimonas_micra	0.001397	0.002315	0.000648	0.001803	0.201	0.484
Selenomonas_massiliensis	0.004733	0.014628	0.035481	0.106281	0.202	0.484
Streptococcus_sobrinus	0.000000	0.000000	0.000938	0.003303	0.208	0.484
Actinomyces_lingnae	0.000480	0.002057	0.000000	0.000000	0.211	0.484
Porphyromonas_gingivalis	0.000803	0.004091	0.007552	0.024178	0.219	0.484
Lancefieldella_parvula	0.021207	0.043661	0.036433	0.042793	0.222	0.484
Firmicutes_oral	0.000747	0.002568	0.000148	0.000487	0.222	0.484
Aggregatibacter_actinomycetemcomitans	0.000000	0.000000	0.001371	0.004984	0.222	0.484
Prevotella_fusca	0.007747	0.024297	0.045424	0.136135	0.224	0.484
Firmicutes_oral	0.001267	0.004872	0.005114	0.014021	0.239	0.484
Streptococcus_mitis	0.012833	0.043008	0.003219	0.007847	0.240	0.484
Pasteurella_multocida	0.572577	0.788315	0.348310	0.572464	0.245	0.484
Bacteroidetes_oral	0.000673	0.002457	0.002462	0.006569	0.245	0.484
Prevotella_dentalis	0.001053	0.004910	0.000000	0.000000	0.250	0.484
Filifactor_alocis	0.010290	0.027939	0.003805	0.010133	0.251	0.484
Capnocytophaga_leadbetteri	0.138683	0.416682	0.046310	0.108467	0.254	0.484
Schaalia_odontolytica	0.196740	0.260163	0.303395	0.361738	0.255	0.484
Rothia_aeria	0.095340	0.111953	0.162948	0.249863	0.256	0.484
Prevotella_oralis	0.000013	0.000073	0.000214	0.000800	0.265	0.484
Staphylococcus_epidermidis	0.000000	0.000000	0.000714	0.002868	0.267	0.484
Streptococcus_suis	0.461487	1.118768	0.223305	0.248364	0.268	0.484
Lactococcus_lactis	0.006810	0.033349	0.000057	0.000262	0.277	0.484
Scardovia_inopinata	0.000233	0.001153	0.000000	0.000000	0.277	0.484
Eubacterium_sulci	0.000627	0.003432	0.004052	0.013839	0.279	0.484
Catonella_genomosp.	0.000763	0.003172	0.000119	0.000376	0.279	0.484
Veillonella_atypica	1.103863	1.593069	1.668486	1.945413	0.280	0.484
Lactobacillus_crispatus	0.000260	0.001298	0.000000	0.000000	0.282	0.484
Porphyromonas_endodontalis	0.018637	0.038559	0.009190	0.024227	0.289	0.484
Streptococcus_pneumoniae	0.483760	0.667648	0.333914	0.316230	0.291	0.484
[Eubacterium]_yurii	0.024457	0.083456	0.007605	0.020010	0.295	0.484
Leptotrichia_wadei	0.230100	0.413233	0.433419	0.818200	0.303	0.484
Dialister_micraerophilus	0.000193	0.001059	0.002086	0.008173	0.304	0.484
Stomatobaculum_longum	0.087607	0.123042	0.125038	0.135481	0.319	0.484
Staphylococcus_aureus	0.016130	0.087445	0.000000	0.000000	0.321	0.484
Neisseria_perflava	0.001857	0.003552	0.018119	0.073149	0.321	0.484
Flavobacteriaceae_bacterium	0.000613	0.002867	0.000081	0.000371	0.323	0.484
Gemella_haemolysins	1.348007	1.304639	1.878419	2.158505	0.323	0.484
Veillonella_parvula	3.256923	3.340880	2.453671	2.406158	0.323	0.484
Prevotella_jejuni	0.151970	0.499842	0.284729	0.444681	0.324	0.484
Prevotella_multiformis	0.000177	0.000968	0.000000	0.000000	0.326	0.484
Ilumatobacter_nonamiensis	0.000040	0.000219	0.000000	0.000000	0.326	0.484
Arcanobacterium_phocae	0.003320	0.018184	0.000000	0.000000	0.326	0.484
Candidatus_Planktophila	0.000017	0.000091	0.000000	0.000000	0.326	0.484
Pseudopropionibacterium_propionicum	0.000007	0.000037	0.000000	0.000000	0.326	0.484
Prevotella_zoogleoformans	0.000087	0.000475	0.000000	0.000000	0.326	0.484
Phocaeicola_abscessus	0.000047	0.000256	0.000000	0.000000	0.326	0.484
Prevotella_maculosa	0.000107	0.000584	0.000000	0.000000	0.326	0.484
Prevotella_saccharolytica	0.000023	0.000128	0.000000	0.000000	0.326	0.484
unidentified_eubacterium	0.000240	0.001315	0.000000	0.000000	0.326	0.484
Campylobacter_rectus	0.000027	0.000146	0.000000	0.000000	0.326	0.484
Arachis_hypogaea	0.000320	0.001753	0.000000	0.000000	0.326	0.484
Desulfovibrio_fairfieldensis	0.000033	0.000183	0.000000	0.000000	0.326	0.484
Alkalibacterium_olivapovliticus	0.000077	0.000420	0.000000	0.000000	0.326	0.484

Lactobacillus_curvatus	0.000660	0.003615	0.000000	0.000000	0.326	0.484
Lactobacillus_gasseri	0.000020	0.000110	0.000000	0.000000	0.326	0.484
Paenibacillus_turicensis	0.000013	0.000073	0.000000	0.000000	0.326	0.484
Moryella_indoligenes	0.000310	0.001698	0.000000	0.000000	0.326	0.484
Oceanivirga_salmonicida	0.000167	0.000913	0.000000	0.000000	0.326	0.484
Acetobacteraceae_bacterium	0.000013	0.000073	0.000000	0.000000	0.326	0.484
Paraburkholderia_kururiensis	0.000247	0.001351	0.000000	0.000000	0.326	0.484
Eogystia_hippophaecolus	0.000040	0.000219	0.000000	0.000000	0.326	0.484
Moraxella_osloensis	0.000127	0.000694	0.000000	0.000000	0.326	0.484
Pseudomonas_fluorescens	0.000030	0.000164	0.000000	0.000000	0.326	0.484
Pseudomonas_mendocina	0.000010	0.000055	0.000000	0.000000	0.326	0.484
Vibrio_litoralis	0.000010	0.000055	0.000000	0.000000	0.326	0.484
Treponema_maltophilum	0.000063	0.000347	0.000000	0.000000	0.326	0.484
Treponema_socranskii	0.000010	0.000055	0.000000	0.000000	0.326	0.484
bacterium_enrichment	0.000147	0.000803	0.000000	0.000000	0.326	0.484
Dialister_invisus	0.148917	0.735108	0.014652	0.039387	0.326	0.484
Lactobacillus_algidus	0.000000	0.000000	0.000433	0.001986	0.329	0.484
Bifidobacterium_aquikefiri	0.000000	0.000000	0.000567	0.002597	0.329	0.484
Rhodococcus_erythropolis	0.000000	0.000000	0.000381	0.001746	0.329	0.484
Kocuria_palustris	0.000000	0.000000	0.000252	0.001157	0.329	0.484
Prevotella_heparinolytica	0.000000	0.000000	0.000052	0.000240	0.329	0.484
Prevotella_genomosp.	0.000000	0.000000	0.000195	0.000895	0.329	0.484
Prevotella_micans	0.000000	0.000000	0.000100	0.000458	0.329	0.484
Bacteroidia_bacterium	0.000000	0.000000	0.000014	0.000065	0.329	0.484
Flavobacterium_aquatile	0.000000	0.000000	0.000095	0.000436	0.329	0.484
Thymallus_thymallus	0.000000	0.000000	0.000010	0.000044	0.329	0.484
Campylobacter_gracilis	0.000000	0.000000	0.000043	0.000196	0.329	0.484
Synura_petersenii	0.000000	0.000000	0.000019	0.000087	0.329	0.484
Anoxybacillus_flavithermus	0.000000	0.000000	0.000086	0.000393	0.329	0.484
Bulleidia_extructa	0.000000	0.000000	0.000200	0.000917	0.329	0.484
Facklamia_languida	0.000000	0.000000	0.000143	0.000655	0.329	0.484
Carnobacterium_inhibens	0.000000	0.000000	0.000029	0.000131	0.329	0.484
Enterococcus_italicus	0.000000	0.000000	0.000962	0.004408	0.329	0.484
Lactobacillus_delbrueckii	0.000000	0.000000	0.001881	0.008620	0.329	0.484
Lactobacillus_kalixensis	0.000000	0.000000	0.000052	0.000240	0.329	0.484
Lactobacillus_salivarius	0.000000	0.000000	0.000852	0.003906	0.329	0.484
Leuconostoc_carnosum	0.000000	0.000000	0.000243	0.001113	0.329	0.484
Streptococcus_downei	0.000000	0.000000	0.000029	0.000131	0.329	0.484
Staphylococcus_capitis	0.000000	0.000000	0.000048	0.000218	0.329	0.484
Staphylococcus_hominis	0.000000	0.000000	0.000133	0.000611	0.329	0.484
iron-reducing_bacterium	0.000000	0.000000	0.000181	0.000829	0.329	0.484
Butyrivibrio_proteoclasticus	0.000000	0.000000	0.001110	0.005084	0.329	0.484
Dorea_formicigenerans	0.000000	0.000000	0.000019	0.000087	0.329	0.484
human_gut	0.000000	0.000000	0.000014	0.000065	0.329	0.484
[Eubacterium]_nodatum	0.000000	0.000000	0.000033	0.000153	0.329	0.484
Selenomonas_artemidis	0.000000	0.000000	0.000033	0.000153	0.329	0.484
Veillonella_denticariosi	0.000000	0.000000	0.000371	0.001702	0.329	0.484
Paracoccus_yeei	0.000000	0.000000	0.000029	0.000131	0.329	0.484
Alcaligenes_faecalis	0.000000	0.000000	0.000076	0.000349	0.329	0.484
Comamonas_aquatica	0.000000	0.000000	0.000090	0.000415	0.329	0.484
Ottowia_oryzae	0.000000	0.000000	0.000033	0.000153	0.329	0.484
Methylotenera_versatilis	0.000000	0.000000	0.000033	0.000153	0.329	0.484
Rhodocyclaceae_bacterium	0.000000	0.000000	0.000210	0.000960	0.329	0.484
Cellvibrio_diazotrophicus	0.000000	0.000000	0.000233	0.001069	0.329	0.484
Cellvibrio_gandavensis	0.000000	0.000000	0.000167	0.000764	0.329	0.484
Proasellus_assaforensis	0.000000	0.000000	0.000052	0.000240	0.329	0.484
Escherichia_coli	0.000000	0.000000	0.000062	0.000284	0.329	0.484
Serratia_plymuthica	0.000000	0.000000	0.007186	0.032929	0.329	0.484
Acinetobacter_baumannii	0.000000	0.000000	0.000033	0.000153	0.329	0.484
Pseudomonas_caeni	0.000000	0.000000	0.000833	0.003819	0.329	0.484
Pseudomonas_syringae	0.000000	0.000000	0.000195	0.000895	0.329	0.484
Synergistetes_bacterium	0.000000	0.000000	0.000043	0.000196	0.329	0.484
bacterium_Ellin516	0.000000	0.000000	0.000033	0.000153	0.329	0.484
Prevotella_oulorum	0.028217	0.059667	0.063881	0.157806	0.333	0.484
TM7_bacterium	0.007727	0.017706	0.004143	0.008025	0.335	0.484
Lactobacillus_acidophilus	0.000003	0.000018	0.000229	0.001047	0.336	0.484
Treponema_denticola	0.000270	0.001405	0.000962	0.003039	0.340	0.484
Prevotella_nanceiensis	0.399763	0.644789	0.254624	0.429936	0.340	0.484
Lactobacillus_reuteri	0.000440	0.002372	0.014843	0.067471	0.340	0.484
Prevotella_buccae	0.000670	0.003267	0.000086	0.000393	0.340	0.484
Lactobacillus_vaginalis	0.000087	0.000475	0.003167	0.014511	0.343	0.485
candidate_division	0.003547	0.015164	0.000852	0.002519	0.347	0.489
Corynebacterium_argentoratense	0.000393	0.002154	0.009510	0.043578	0.350	0.490
Bifidobacterium_longum	0.003297	0.017720	0.043048	0.190522	0.352	0.491
Peptostreptococcaceae_bacterium	0.003837	0.020864	0.000252	0.001157	0.355	0.494
Lactobacillus_fermentum	0.001090	0.005970	0.018381	0.084232	0.359	0.496

Eubacterium_saphenum	0.000017	0.000091	0.000210	0.000960	0.370	0.508
Lactobacillus_helveticus	0.001537	0.008417	0.016733	0.076134	0.373	0.511
TM7_phylum	0.180180	0.288289	0.262290	0.351335	0.383	0.521
Mobiluncus_curtisii	0.000120	0.000657	0.000705	0.002964	0.384	0.521
Johnsonella_ignava	0.000113	0.000584	0.000019	0.000087	0.391	0.527
Shuttleworthia_satelles	0.000413	0.002264	0.001176	0.003629	0.400	0.537
Capnocytophaga_granulosa	0.002577	0.005922	0.001381	0.004207	0.403	0.539
Corynebacterium_diphtheriae	0.004487	0.010887	0.022562	0.099777	0.418	0.555
TM7_phylum	0.002090	0.009772	0.000595	0.001895	0.420	0.555
TM7_phylum	0.071310	0.111738	0.110252	0.199938	0.425	0.555
Firmicutes_oral	0.000063	0.000268	0.000352	0.001615	0.426	0.555
Lactobacillus_amylovorus	0.000520	0.002848	0.002938	0.013464	0.427	0.555
Lachnobacterium_bovis	0.001063	0.005824	0.000195	0.000895	0.428	0.555
Candidatus_Saccharibacteria	0.001040	0.003821	0.002276	0.006309	0.429	0.555
Haemophilus_sputorum	0.023457	0.040435	0.070248	0.265483	0.432	0.556
Lachnospiraceae_bacterium	0.003527	0.008655	0.002129	0.004008	0.443	0.566
Leptotrichia_shahii	0.018703	0.052895	0.038590	0.108573	0.444	0.566
Dialister_pneumosintes	0.001790	0.006813	0.003729	0.010017	0.446	0.567
Neisseria_oralis	0.008497	0.023947	0.016848	0.045879	0.451	0.570
Megasphaera_elsdenii	0.000057	0.000310	0.000219	0.001004	0.480	0.605
Campylobacter_concisus	0.027087	0.028504	0.021633	0.027375	0.495	0.618
Alloprevotella_tannerae	0.088927	0.284350	0.050419	0.094866	0.495	0.618
Prevotella_pleuritidis	0.006747	0.022648	0.003567	0.009849	0.499	0.619
Prevotella_salivae	0.535690	0.706516	0.751957	1.326631	0.501	0.619
Streptococcus_oralis	0.005743	0.011762	0.008019	0.012613	0.518	0.638
Snodgrassella_alvi	0.000207	0.001132	0.000067	0.000306	0.523	0.642
Lactobacillus_iners	0.000363	0.001990	0.000124	0.000396	0.526	0.642
Tannerella_forsythia	0.012717	0.032121	0.008062	0.020495	0.531	0.643
Eikenella_corrodens	0.000123	0.000604	0.000048	0.000218	0.532	0.643
Streptococcus_australis	0.010840	0.029973	0.006714	0.016896	0.535	0.643
Streptococcus_gordonii	0.034577	0.042083	0.027752	0.035642	0.536	0.643
Lachnoanaerobaculum_umeaense	0.261820	0.440938	0.520000	1.904735	0.548	0.653
Treponema_refringens	0.000013	0.000073	0.000005	0.000022	0.549	0.653
Gemella_sanguinis	0.063197	0.081760	0.080300	0.111594	0.553	0.655
Actinomyces_dentalis	0.000020	0.000081	0.000052	0.000240	0.557	0.658
Leptotrichia_hongkongensis	0.000443	0.001303	0.000838	0.002894	0.564	0.662
Streptococcus_salivarius	9.355673	9.977574	11.025995	10.421298	0.570	0.666
Prevotella_intermedia	0.162933	0.306408	0.128262	0.142820	0.591	0.689
Bacteroidales_oral	0.000693	0.002657	0.000367	0.001680	0.594	0.689
Cardiobacterium_valvarum	0.004517	0.007016	0.003262	0.009182	0.601	0.692
Capnocytophaga_ochracea	0.072567	0.235687	0.045290	0.133491	0.602	0.692
Prevotella_baroniae	0.000297	0.001155	0.000167	0.000606	0.604	0.692
Porphyromonas_pasteri	0.832690	1.094066	0.660667	1.228903	0.610	0.696
Neisseria_bacilliformis	0.001420	0.004222	0.000929	0.002782	0.619	0.704
Leptotrichia_hofstadii	0.004440	0.008972	0.003500	0.004943	0.634	0.718
Leptotrichia_trevisanii	0.000373	0.001512	0.000210	0.000960	0.639	0.719
[Eubacterium]_brachy	0.001553	0.007195	0.002586	0.008090	0.642	0.719
Streptococcus_anginosus	0.026693	0.098808	0.046890	0.178942	0.642	0.719
Gracilibacteria_bacterium	0.000187	0.000660	0.000124	0.000342	0.659	0.730
Slackia_exigua	0.000227	0.001242	0.000114	0.000524	0.660	0.730
Solobacterium_moorei	0.264917	0.350750	0.224710	0.295646	0.660	0.730
Prevotella_enoeca	0.000040	0.000154	0.000024	0.000109	0.663	0.730
TM7_phylum	0.000873	0.004783	0.000457	0.002095	0.675	0.741
Candidatus_Aquiluna	0.000053	0.000218	0.000095	0.000436	0.688	0.752
Streptococcus_peroris	0.000797	0.004364	0.001224	0.003692	0.708	0.771
Rothia_mucilaginosa	5.253723	8.216198	4.557562	5.180235	0.713	0.773
Leptotrichia_buccalis	0.209983	0.341634	0.176981	0.321077	0.727	0.785
Kingella_denitrificans	0.000263	0.000952	0.000190	0.000641	0.745	0.802
Actinomyces_viscosus	0.000270	0.001479	0.000171	0.000786	0.759	0.814
Anaeroglobus_geminatus	0.000140	0.000767	0.000090	0.000415	0.768	0.820
Flavobacterium_branchiophilum	0.000260	0.000865	0.000190	0.000873	0.780	0.828
Howardella_ureilytica	0.000123	0.000676	0.000186	0.000851	0.781	0.828
Coregonus_clupeaformis	0.000103	0.000566	0.000152	0.000698	0.791	0.836
Candidatus_Saccharimonas	0.000207	0.000787	0.000157	0.000720	0.817	0.860
Streptococcus_sanguinis	0.083020	0.153738	0.094452	0.198100	0.826	0.866
Selenomonas_sputigena	0.027787	0.074025	0.024543	0.044906	0.847	0.884
Cryptobacterium_curtum	0.000077	0.000420	0.000100	0.000458	0.854	0.889
Prevotella_nigrescens	0.020547	0.040130	0.018371	0.047016	0.864	0.896
Prevotella_veroralis	0.023803	0.128774	0.019129	0.087658	0.878	0.907
Cardiobacterium_hominis	0.002900	0.004744	0.003205	0.008532	0.883	0.909
Fusobacterium_nucleatum	0.511770	0.479372	0.487657	0.717682	0.894	0.917
Selenomonas_flueggei	0.001467	0.004997	0.001595	0.004535	0.924	0.943
Streptococcus_cristatus	0.027300	0.052597	0.028386	0.032443	0.928	0.943
Actinomyces_graevenitzii	0.722543	1.084772	0.750290	1.102593	0.929	0.943
Alloprevotella_rava	0.197623	0.521587	0.184862	0.656306	0.941	0.951
Kingella_oralis	0.001470	0.005333	0.001400	0.003545	0.955	0.962

Prevotella_melaninogenica	7.614003	7.171547	7.633257	6.417578	0.992	0.996
Peptostreptococcaceae_bacterium	0.002140	0.011721	0.002148	0.009030	0.998	0.998

Supplementary table 4: Comparison of the study results to a historic control group of healthy children

**(Abdulhaq et al., 2021)**<br><br>**N=38 neurotypical controls**	**Study participants CKD patients**<br><br>**N=30 CKD patients**	**(Abdulhaq et al., 2021)**<br><br>**N=38 neurotypical controls**	**Study participants CKD patients**<br><br>**N=30 CKD patients**
**Genera**<br><br>Average tongue microbiome composition by genus	**Genera**<br><br>Average tongue microbiome composition by genus	**Species**<br><br>Average tongue microbiome composition by species	**Species**<br><br>Average tongue microbiome composition by species
Prevotella<br>(15.3%)	Prevotella<br>(12.3%)	Haemophilus parainfluenza<br>(7.5%)	Haemophilus_parainfluenzae<br>(2.8%)
Streptococcus<br>(14.8%)	Streptococcus<br>(28.4%)	Rothia mucilaginosa<br>(7.2%)	Rothia_mucilaginosa<br>(11.4%)
Leptotrichia<br>(10.5%)	Leptotrichia<br>(0.8%)	Prevotella<br>Melaninigenica<br>(6.2%)	Prevotella_melaninogenica<br>(20.2%)
Veillonella<br>(7.9%)	Veillonellaceae<br>(6,8%)	Neisseria flavescens/subflava<br>(5.8%)	-
Haemophilus<br>(7.6%)	Haemophilus<br>(3.4%)		Neisseria_meningitidis<br>(21.1%)
Rothia<br>(7.3%)	Rothia<br>(7,3%)	Porphyromonas sp. HOT 417<br>(5.5%)	Porphyromonas_pasteri<br>(1.8%)
Neisseria<br>(6.5%)	Neisseria<br>(15,8%)	Veillonella parvula<br>(5.2%)	Veillonella_parvula<br>(6.7%)
Porphyromonas<br>(5.7%)	Porphyromonas<br>(1.2%)	-	Veillonella_atypica<br>(2%)
Fusobacterium<br>(5.0%)	Fusobacterium<br>(1.6%)	Leptotrichia sp. 417<br>(4.9%)	-
Actinomyces<br>(4.3%)	Actinomyces<br>(2.2%)	Fusobacterium periodonticum<br>(4.8%)	Fusobacterium_periodonticum<br>(2.9%)
Granulicatella<br>(2.6%)	-	Prevotella histicola<br>(3.7%)	-
-	Gemella<br>(1.8%)	Streptococcus<br>Parasanguinis II<br>(2.8%)	Streptococcus_parasanguinis<br>(2.2%)
		Streptococcus infantis<br>(2.7%)	-
		-	Streptococcus_<br>Salivarius<br>(23.7%)
		Granulicatella adiacens<br>(2.3%)	-
		Leptotrichia sp. HOT 215<br>(1.7%)	-
		-	Gemella_haemolysans<br>(3.3%)
		-	Actinomyces_graevenitzii<br>(1.4%)

Reference of the historic controls used for comparison in Supplementary table 4: Abdulhaq, A., Halboub, E., Homeida, H. E., Kumar Basode, V., Ghzwani, A. H., Zain, K. A., . . . Al-Hebshi, N. N. (2021). Tongue microbiome in children with autism spectrum disorder. J Oral Microbiol, 13(1), 1936434. doi:10.1080/20002297.2021.1936434
